# Hot spots of unseen fishing vessels

**DOI:** 10.1126/sciadv.abq2109

**Published:** 2022-11-02

**Authors:** Heather Welch, Tyler Clavelle, Timothy D. White, Megan A. Cimino, Jennifer Van Osdel, Timothy Hochberg, David Kroodsma, Elliott L. Hazen

**Affiliations:** ^1^ Institute of Marine Sciences, University of California Santa Cruz, Santa Cruz, CA, USA 95064.; ^2^ Environmental Research Division, Southwest Fisheries Science Center, National Marine Fisheries Service, National Oceanic and Atmospheric Administration, Monterey, CA, USA 93940.; ^3^ Global Fishing Watch, Washington, DC, USA 20036.; ^4^ Hopkins Marine Station, Stanford University, Pacific Grove, CA 93950, USA.

## Abstract

Illegal, unreported, and unregulated (IUU) fishing incurs an annual cost of up to US$25 billion in economic losses, results in substantial losses of aquatic life, and has been linked to human rights violations. Vessel tracking data from the automatic identification system (AIS) are powerful tools for combating IUU, yet AIS transponders can be disabled, reducing its efficacy as a surveillance tool. We present a global dataset of AIS disabling in commercial fisheries, which obscures up to 6% (>4.9 M hours) of vessel activity. Disabling hot spots were located near the exclusive economic zones (EEZs) of Argentina and West African nations and in the Northwest Pacific, all regions of IUU concern. Disabling was highest near transshipment hot spots and near EEZ boundaries, particularly contested ones. We also found links between disabling and location hiding from competitors and pirates. These inferences on where and why activities are obscured provide valuable information to improve fisheries management.

## Teaser

Global analysis of intentional automatic identification system disabling provides insights to aid ocean governance.

## INTRODUCTION

Monitoring human activity at sea remains a serious challenge. Over the past decades, industries such as fishing, shipping, and mineral resource exploration have expanded beyond national jurisdictions into the high seas, while tools to monitor and regulate these activities have lagged behind (*1*). This lack of effective monitoring combined with a fragmented legal framework in which monitoring requirements differ across nations and international waters (*2*) has allowed illegal, unreported, and unregulated (IUU) fishing to operate on a large, systemic scale (*3*). Global economic losses from IUU fishing are valued to be between US$10 billion and US$25 billion annually, with one in every five wild-caught fish harvested illegally or unreported (*3*). Labor abuses have been linked to IUU fishing, violating human rights through forced labor and trafficking (*4*, *5*).

The ability to publicly monitor fishing fleets and fisher behavior has been revolutionized by leveraging the shipboard automatic identification system (AIS), which was created as a collision avoidance tool (*6*). AIS data have been used to produce unprecedented views of global fishing activity and the corporate actors involved in it (*7*, *8*), assess the impacts of conservation actions such as marine protected areas (MPAs) (*6*, *9*, *10*), and reveal illegal fishing and insight into potential forced labor activities (*11*, *12*). However, the utility of AIS as a monitoring tool is impeded by vessels intentionally disabling their AIS devices, effectively obscuring their activities from public scrutiny. Until now, we have not been able to quantify the scale, spatial footprint, or drivers of intentional AIS disabling.

AIS devices are not universally mandated, nor are vessels always required to keep their devices on (*13*), and as such, intentional disabling of AIS may signal both legal and illegal activities. In some cases, fishing vessels are exempted from AIS requirements because fishing locations are considered confidential (*13*). Vessels might also choose to disable their devices to avoid hostile interactions in waters prone to piracy (*14*). The disabling of AIS devices can obscure illegal activities, such as unauthorized fishing activity in exclusive economic zones (EEZs) and MPAs, or unauthorized transshipments, in which catch from fishing vessels is off-loaded to refrigerated cargo vessels at sea (*15*). Transshipment can reduce fisheries’ operating costs and allow catch to be transported more efficiently (*16*, *17*), yet when poorly monitored, it can provide a means to launder illegally caught seafood into the market and, in some fisheries, has been linked to IUU fishing, forced labor, and human trafficking (*18*, *19*).

Although AIS disabling may obscure vessel behavior at sea, we can use these events as a data source to direct research and improve management of our global fisheries. Here, we present the first global dataset and analysis of suspected AIS disabling in commercial fisheries. We first develop a rule-based classification model to identify which gaps in AIS transmission are likely to be caused by intentional disabling, and reveal the locations, flag states, and gear types that have the most fishing vessel activity obscured by disabling. We then use a popular machine learning method called boosted regression trees to identify the primary drivers of suspected AIS disabling from a suite of potential drivers related to fishing ground quality, piracy, transshipment, and jurisdictional boundaries. This work draws attention to the areas and fleets in which AIS disabling may compromise the utility of AIS as a monitoring tool and where stricter AIS requirements and enforcement may support improved fisheries management.

## RESULTS

### A global picture of AIS disabling

The Global Fishing Watch AIS dataset of fishing vessel activity includes more than 3.7 billion AIS messages from fishing vessels between 2017 and 2019. Within this dataset, we identified more than 55,000 suspected intentional disabling events in waters greater than 50 nautical miles from shore. More than 40% of fishing vessels in these waters had suspected disabling events, obscuring up to 6% (>4.9 M hours) of fishing vessel activity (Table 1).

**Table 1.** Dimensions of the suspected disabling dataset by gear type and flag state. The lower bound in the ranges for the total time lost and fraction of time lost to AIS disabling events result from capping suspected disabling events at 2 weeks. The upper bound includes all disabling events.

		**AIS disabling events (*n*** **)**	**Time lost to AIS disabling events (days)**	**Fraction of time lost to AIS disabling events (%)**
**Gear type**	Drifting longlines	18,641	32,826–83,202	2.0–4.6
	Squid jiggers	16,021	25,602–39,524	5.0–7.2
	Tuna purse seines	8620	19,945–44,735	10.7–20.6
	Trawlers	7913	14,980–22,823	3.5–5.0
	Other gear types	4173	7446–16,423	2.0–4.0
**Flag**	China	15,624	23,463–45,440	3.0–5.4
	Chinese Taipei	12,867	23,170–43,872	3.8–6.3
	Spain	4100	10,058–23,881	6.5–13.8
	United States	3543	8265–16,822	4.7–8.3
	Other flags	19,234	35,844–76,693	2.5–5.0
	**All vessels**	**55,368**	**100,800–206,707**	**3.2–6.0**

**Fig. 1.**
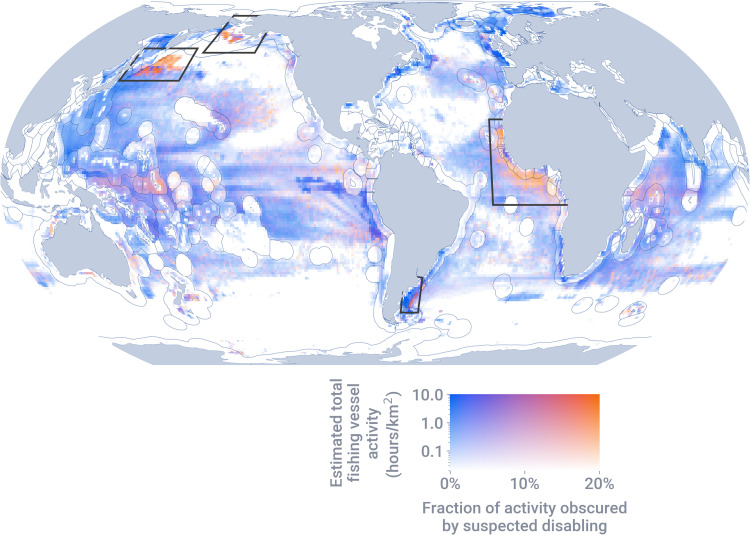
Estimated total fishing vessel activity and the fraction of this activity obscured by suspected disabling events in areas with sufficient satellite reception quality (>10 positions/day). Areas with the highest fishing vessel activity and the highest fraction of activity obscured by disabling occur in three regions of IUU concern: near Argentina and West Africa and in the Northwest Pacific (black boxes). In contrast, fisheries in waters near Alaska, USA are some of the most intensively managed in the world.

We estimated the fraction of fishing vessel activity obscured by AIS disabling events to identify disabling hot spots, i.e., locations with both high fishing vessel activity and a high fraction of this activity obscured by disabling (Fig. 1). More than 40% of the total hours lost to suspected disabling occurred across four hot spots: the Northwest Pacific (13%), adjacent to the EEZs of Argentina (16%) and West African nations (8%), and near Alaska, USA (3%).

**Fig. 2.**
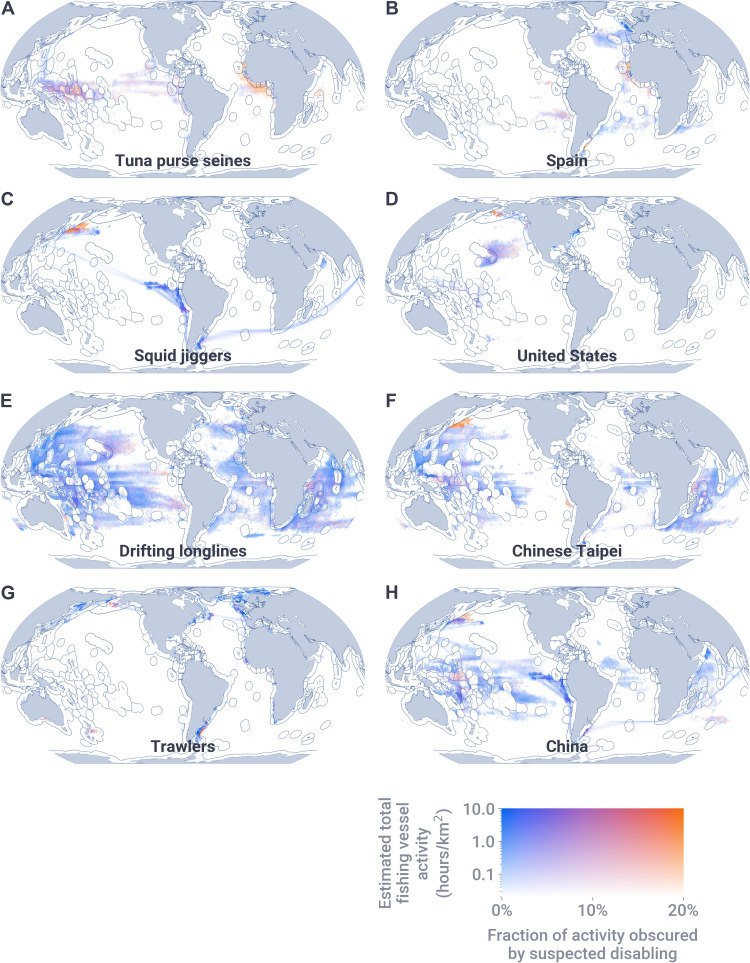
Estimated total fishing vessel activity and the fraction of this activity obscured by suspected disabling events. Panels **A**, **C**, **E**, and **G** show the gear types with the most time obscured by disabling events; panels **B**, **D**, **F**, and **H** show the flag states with the most time obscured by disabling events. Only areas with sufficient satellite reception quality (>10 positions/day) are shown.

Four gear types made up 96% of time lost to disabling events in waters more than 50 nautical miles from shore (Fig. 2, A, C, E, and G). Tuna purse seines had the highest fraction of vessel activity obscured by disabling events (up to 21%), followed by squid jiggers (up to 7%), and drifting longlines and trawlers (both up to 5%; Table 1). Vessels flagged to four key nations made up 82% of time lost to suspected disabling events in waters more than 50 nautical miles from shore (Fig. 2, B, D, F, and H). Of these nations, Spain had the highest fraction of vessel activity obscured by disabling events (up to 14%), followed by the United States (up to 8%), Chinese Taipei (up to 6%), and China (up to 5%; Table 1). In terms of absolute hours, drifting longlines and China had the most total time lost to suspected disabling across gear types and flag states, respectively (Table 1). AIS usage is biased toward vessels flagged to upper- and middle-income countries (*20*); thus, these results, in part, reflect who uses AIS (higher AIS usage leads to high opportunity to disable AIS).

### Drivers of suspected AIS disabling

We used boosted regression trees to understand how the locations where suspected disabling occurs differ from the locations where fishing occurs. Model presences were locations with suspected disabling, and model absences were locations with fishing activity and no suspected disabling events. We built individual boosted regression tree models for the four dominant gear types (squid jiggers, trawlers, tuna purse seines, and drifting longlines) and a full model that included all suspected disabling events (i.e., the four gear types listed above and additional gears such as gillnet and troll). All models had high explained deviance and predictive skill [32.7% mean explained deviance and 0.83 mean area under the receiver operator characteristic curve (AUC); table S6], indicating a strong capacity to discriminate between locations with disabling events (presences) and locations with fishing activity and no disabling events (absences).

**Fig. 3.**
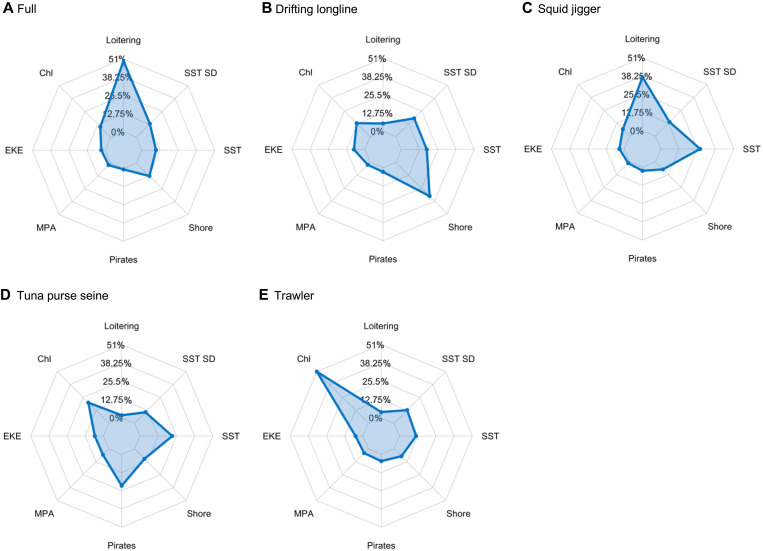
The relative importance of drivers in suspected disabling models. Panels show the relative importance of drivers for the full (**A**) and gear-specific (**B** to **E**) models. For each model, the importance of drivers sums to 100%. Chl, chlorophyll-a; SST, sea surface temperature; SST SD, the variability of SST across time; Pirates, distance to reported piracy events; MPA, distance to MPA; EKE, eddy kinetic energy; Shore, distance to shore.

**Fig. 4.**
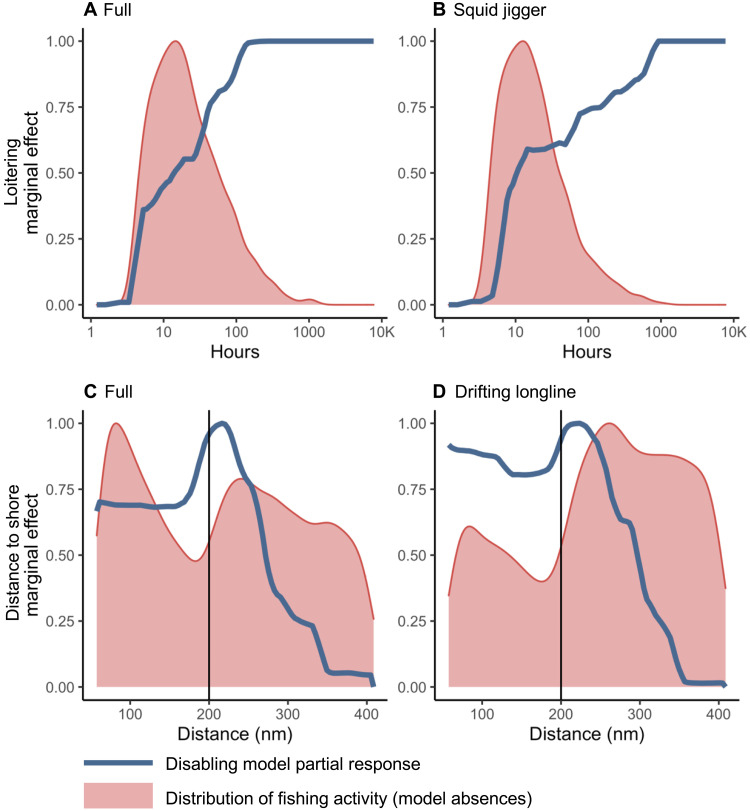
Partial dependence plots for the suspected disabling models. Panels show the partial dependence plots for loitering activity (**A** and **B**) and distance to shore (**C** and **D**), for the full (A and C), squid jigger (B), and drifting longline (D) models. Each plot shows the model marginal response (blue) and distribution of model absences (fishing activity in locations with no disabling; red).

We evaluated the relative importance of drivers to understand why disabling occurs in certain areas and not others (Fig. 3). Loitering by transshipment vessels (a proxy for potential transshipment events) was the most important driver in the full and squid jigger models (relative importance of 49 and 37%, respectively; Fig. 3). As loitering activity increased, the occurrence of suspected disabling in the full and squid jigger models increased (Fig. 4, A to C), particularly in areas with 1000 to 10,000 hours of loitering. In contrast, fishing activity was distributed in areas with 100 hours or less of loitering (Fig. 4, A to C). Furthermore, more than half of the disabling events by squid jiggers were close enough to loitering refrigerated cargo vessels to be able to undertake a transshipment (fig. S21). In contrast, loitering was less important in suspected disabling models for drifting longlines, tuna purse seines, and trawlers (Fig. 3).

Distance to shore was the most important driver in the suspected disabling model for drifting longlines (33% relative importance) and was the third most important in the full model (13% relative importance; Fig. 3). The drifting longline and full models showed peaks in disabling occurrence at or just outside 200 nautical miles from shore, with occurrence decreasing further offshore (Fig. 4, C and D). Peaks in occurrence at 200 nautical miles were also apparent in models for squid jiggers and tuna purse seines (fig. S23, A and B), although relative importance was lower (7 and 10%, respectively; Fig. 3). This distance marks the location of most EEZ boundaries. In contrast, fishing activity for the drifting longline and full models was bimodally distributed, with peaks both inshore and beyond 200 nautical miles (Fig. 4, C and D).

The spike in suspected disabling at EEZ boundaries is concerning because it suggests that some of these events may obscure unauthorized boundary crossings. Notably, more than 96% of EEZ-adjacent disabling events occurred next to foreign EEZs. However, many global fishing hot spots are located just outside EEZ boundaries; thus, this pattern may be partially explained by the increased levels of fishing activity in these areas. We compared the amounts of suspected disabling and fishing activity near EEZs to investigate whether there was more disabling than would be expected by the amount of fishing activity. Waters adjacent to the EEZs of Argentina, Russia, and Peru had higher percentages of disabling events compared to fishing activity, with more than 47% of all disabling events adjacent to the Argentinean EEZ (fig. S22A). Across all flag states, China and Spain had higher percentages of suspected disabling events adjacent to EEZs relative to their fishing activity (fig. S22B). While only a small percentage (11%) of EEZs have contested boundaries (overlapping claims from opposing nations, e.g., the Kuril Islands EEZ, claimed by both Japan and Russia), disabling events were twice as likely within or adjacent to these EEZs compared to fishing activity. These results suggest that, in some key locations and fleets, the high amount of suspected disabling near EEZ boundaries cannot be explained by the amount of fishing activity alone.

Chlorophyll and piracy were also important drivers of disabling. Chlorophyll was the most important driver in the trawler model (50%), followed by tuna purse seines, drifting longline, and full models (relative importance of 20, 13, and 10%, respectively) (Fig. 3). Marginal effect plots indicated increased disabling in areas with higher productivity (fig. S24, A to D), whereas fishing was distributed across areas of more moderate productivity. For tuna purse seines, piracy had a relative importance of 22% (Fig. 3D) due to a high concentration of disabling events in areas of high pirate activity near West Africa and in the Arabian Sea (figs. S2C and S19D). The marginal effects indicated that the occurrence of tuna purse seine disabling decreases with increasing distance from high pirate activity areas, whereas the distribution of fishing activity showed a lack of a relationship with distance (fig. S24E).

## DISCUSSION

In this study, we examined suspected disabling events, characterized by gaps in AIS transmissions in which we have the most confidence that vessels intentionally disabled their transponders. An estimated 50 to 80% of fishing operations occurring more than 100 nautical miles from shore are by vessels with AIS (*7*), and a majority of the effort on the high seas is by vessels broadcasting AIS (*21*). Our suspected disabling dataset illuminates an additional 4.9 M hours (6%) of fishing vessel activity by vessels with AIS, allowing inference on activities at sea that have previously been opaque to the public.

Notably, the disabling hot spots in the Northwest Pacific and adjacent to the EEZs of Argentina and West African nations have been previously deemed to be areas of IUU concern (*17*, *22*–*24*): All areas contain rich fishing grounds and limited management oversight—a formula that may embolden vessels to engage in IUU activities. One study found that more than half of the vessels flagged to China operating in the Northwest Pacific had suspected disabling events and estimated that more than half of their chub mackerel catch was unreported (*22*). In the high seas adjacent to Argentina, transshipments are potentially subject to fewer regulations than national waters. It is hypothesized that some nonbroadcasting “dark” fleets fish without authorization within Argentinian waters and off-load catch to high seas refrigerated cargo vessels, thus avoiding visiting ports to off-load illegal catch (*17*). As West Africa suffers annual economic losses of up to US$2.3 billion because of illegal fishing, including authorized incursions of industrial fleets into national waters, AIS disabling that reduces monitoring capabilities in this region is particularly alarming (*23*, *24*).

Vessels outfitted with government-mandated vessel monitoring systems (VMS) may remain trackable by flag states regardless of AIS use, although access to VMS data is often tightly restricted by national governments; thus, AIS disabling may prevent other governments, coastal states, and nearby ships from detecting such vessels. The disabling hot spots in the Northwest Pacific and adjacent to the EEZs of Argentina and West African nations are largely the products of distant water fleets operating in foreign and international waters (Fig. 2), and it is unlikely that local authorities have access to VMS information from these vessels. In contrast, the hot spot in U.S. waters offshore of Alaska was caused by disabling in U.S. trawlers (Fig. 2): a fleet that is monitored by U.S. authorities using VMS. This area is considered one of the most intensely managed regions in the world (*25*), and disabling in this region likely indicates location hiding of high-quality fishing grounds from competitors (unlike AIS, VMS cannot be legally disabled, nor can VMS messages be received by competitors).

Disabling events were common in areas with high transshipment activity, while fishing activity occurred in areas with lower transshipment activity. These results indicate a desire to have these possibly unauthorized transfers obscured from public view. This pattern was particularly pronounced for squid jiggers, which are known to operate in areas of high loitering activity (*17*), and have relatively little management oversight compared to other gear types and target species (e.g., tunas). For tuna purse seines, loitering may have less importance because transshipment regulations and fisheries observer requirements are generally stricter for this gear type than for other gear types (*26*, *27*).

Disabling events were concentrated in waters adjacent to EEZ boundaries, while fishing occurred primarily inshore or in high seas outside EEZ boundaries. Disabling events were most likely to occur adjacent to foreign and disputed EEZs, suggesting that some of these events may indicate vessels disabling their transponders before entering unauthorized locations to fish illegally. Furthermore, the disproportionate concentration of disabling within and near EEZs with contested boundaries suggests that the political conflicts in these regions may create managerial blind spots, which could make IUU activities more profitable and less likely to be prosecuted. However, the lack of transparency in fisheries’ access agreements (arrangements in which coastal countries allow foreign nations to fish within their EEZs) obscures which disabling events may warrant further attention.

While it appears that AIS devices may be disabled to obscure unauthorized behaviors such as transshipments and border crossings, there was also evidence that disabling may occur for legal activities. Chlorophyll was an important driver of disabling in trawlers and tuna purse seines. This pattern suggests that these gear types may typically disable their AIS devices to hide the locations of productive fishing grounds from competitors, although a variety of motivations are possible (e.g., avoiding competition and masking IUU activity or impacts on sensitive habitats). Trawling activity is far more spatially concentrated than other gear types because it is restricted to continental shelves, making competition particularly impactful in this gear type (*7*, *28*). Piracy was the most important driver of tuna purse seine disabling, indicating that one of the reasons tuna purse seines disable their AIS devices is likely to hide their positions in dangerous waters. While these patterns do not suggest IUU, intense fishing pressure has led to overexploitation and population decline in many commercial stocks (*29*). Furthermore, fisheries interactions such as bycatch, entanglement, or ship strike threaten vulnerable and protected species (*30*–*32*). Information on AIS disabling provides insights into the scale and locations of hidden fishing activity and where increased monitoring can help subvert these negative ecosystem impacts.

### Disabling case study

**Fig. 5.**
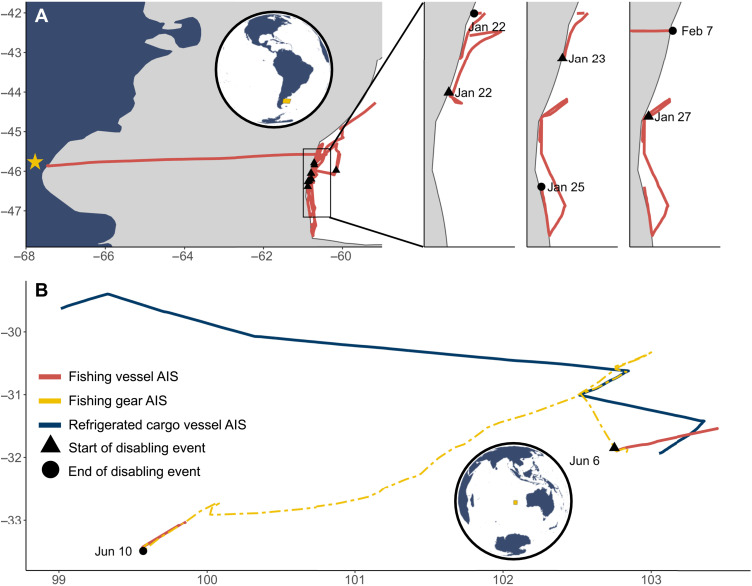
Two examples of fishing vessels disabling their AIS devices to obscure their activities from oversight. (**A**) The *Oyang 77* disabled its AIS device nine times (the last three of which are shown in insets) adjacent to the Argentinean EEZ (gray shading) before being apprehended by Argentinian coast guard and escorted back to port (yellow star). (**B**) A fishing vessel disabled its vessel-based AIS device but left its gear-mounted device broadcasting, showing transhipment with a refrigerated cargo vessel during the disabling event.

The legality of disabling events is often difficult to discern because policies on AIS usage vary by region, regional fisheries management organization (RFMO), flag state, and vessel size. This complex governance creates an intricate patchwork of AIS regulations that makes it challenging to identify which disabling events are, by themselves, truly illegal. Although we are unable to confirm which disabling events are illegal, we have demonstrated strong links between suspected disabling and behaviors of IUU concern, specifically potentially unauthorized border crossings and transshipments. The *Oyang 77* disabled its AIS device nine times adjacent to the Argentinean EEZ before being apprehended by the coast guard and charged with illegally fishing within Argentina’s waters (Fig. 5A) (*33*). Similarly, the *Lu Rong Yuan Yu 668* disabled its AIS device twice on the high seas beyond Argentina’s EEZ and was eventually captured by the coast guard for illegally fishing within Argentina’s waters (*34*).

A clear example of transshipment occurring during a disabling event was made visible because of a gear-mounted AIS device (presumably used to reduce loss of expensive fishing gear) that was broadcasting during a suspected disabling event (Fig. 5B). During this event, the fishing vessel disabled its vessel-based AIS but left its gear-mounted AIS broadcasting before an encounter with a refrigerated cargo vessel and a transshipment event confirmed by the vessel’s flag state. Although both vessels involved in this particular transshipment event were properly authorized by the relevant RFMOs to transship in the region, it reveals that transshipment events do take place during disabling events. In tandem, the importance of loitering and distance to EEZ to our models suggests a potential linkage between AIS disabling and IUU in some regions.

IUU fishing activities remain a leading threat to human rights, marine ecosystems, and the global economy (*3*, *11*, *35*). Because of the severity and extent of these impacts, combating IUU fishing has become an international priority. Both the United Nations Sustainable Development Goals and the Convention on Biological Diversity included targets to eliminate or reduce IUU activities by 2020 (*36*). So far, these goals have proved elusive because of the challenge of surveilling, let alone policing, nefarious acts that are committed in remote waters by actors who do not want their behaviors observed. Our models for suspected disabling had strong predictive power on previously unseen data (table S6), indicating that our approach could be used to identify areas of disabling concern as previously unseen data become available. Such prediction systems could be used to position at-sea or airborne enforcement and surveillance or applied in conjunction with information on risky ports (*37*) to guide and focus IUU inspections required by the Port State Measures Agreement (*38*). Goals to eliminate or reduce IUU fishing have, thus far, been unattainable because of a paucity of data on who is involved in IUU activities and where and why these behaviors occur. Ironically, it is the absence of AIS data itself that contains a wealth of information and can serve as a valuable tool in the data-deficient fight against IUU fishing activity.

## METHODS

### AIS dataset

We acquired more than 28 billion AIS messages from 2017 to 2019 from Global Fishing Watch (GFW) from Spire and Orbcomm. This time series was bound by the 2017 launch of Spire satellites (see section S1), providing more data points on AIS usage, and available AIS data for 2017–2019 from exactEarth for use in model validation (see section S3.2). Using a convoluted neural network (*7*), the AIS messages were processed to identify fishing versus nonfishing events and gear type. The data were summed across the time series, aggregated into quarter degree rasters (the coarsest resolution of the environmental datasets) and summarized using three fields related to fleet behavior: vessel days (fig. S1A), fishing days (fig. S1B), and suspected AIS disabling events (fig. S2).

### Definition of disabling events

Gaps in AIS data occur for several technical reasons unrelated to intentional disabling: signal interference in crowded waters, spatial variability of terrestrial reception, spatial and temporal variability of satellite reception, and dropped signals as vessels move from terrestrial coverage to areas of poor satellite reception. We therefore restricted our analysis to waters further than 50 nautical miles from shore to control for signal interference in crowded near-shore waters and dropped signals during the terrestrial-satellite receiver changeover (see section S1.1). To address the spatial variability of satellite reception, we produced maps of observed reception quality for class A and B AIS devices at 1°, calculated as the average number of AIS messages received by satellites per vessel per day (fig. S3 and section S2.1). We also produced maps of predicted reception quality for class A and B AIS devices at 0.25°, using a radial basis function for interpolation (fig. S4 and section S2.2). This provided a finer-scale estimate of the spatial variability in reception quality. We restricted our analysis to AIS gap events in areas with a predicted reception quality of more than 10 positions/day, a threshold above which the subsequent choice of an optimal model to identify disabling events was unaffected (section S3.4). To address the temporal variability of satellite reception, we excluded gap events shorter than 12 hours (see section S2.3). Under this threshold, the number of satellites overhead varies substantially (fig. S7), resulting in AIS gap events that are not indicative of intentional disabling.

The above filters (AIS gap events of at least 12 hours in waters more than 50 nautical miles from shore, in areas with more than 10 pings/day) excluded the gap events in which we have the least confidence in being able to properly classify them as intentional or unintentional disabling. We used a rule-based classification model on the remaining gaps to further restrict gap events to those that we have the most confidence due to intentional disabling. To identify the highest-performing rule-based classification model, we tested a series of minimum thresholds for the predicted reception quality and ping (broadcast) rates over several intervals before each gap event (see section S3.1) against a labeled test set of intentional AIS disabling events from exact Earth (see section S3.2). Model selection was performed using F0.5 scores generated during repeated *k*-fold cross-validation (see section S3.3). The final model—12 hours before with a position ping rate threshold, *k*, of 14—had an F0.5 score of 0.739. This model performs well at limiting false positives with a false-positive rate of 3.72% and a precision of 0.86 (table S2, fig. S12, and section S3.4). We applied this model to generate a final AIS disabling dataset of 55,368 events including 5269 distinct Maritime Mobile Service Identity (MMSI) from 101 flag states.

To quantify the scale of the problem caused by AIS disabling events, we spatially allocated the time between disabling event start and end using both a linear interpolation and a rasterized probability method (see section S5 and figs. S15 to S18). Because the two methods performed similarly for disabling events shorter than 2 weeks but deviate for longer disabling events (table S3), we calculate statistics for both (Table 1), but only map the fraction of fishing activity obscured using disabling events shorter than 2 weeks (Figs. 1 and 2). We calculated the fraction of time lost to disabling events as the time lost to disabling events divided by the time at sea for all fishing vessels in the study area (see section S5.3).

### Boosted regression trees

**Fig. 6.**
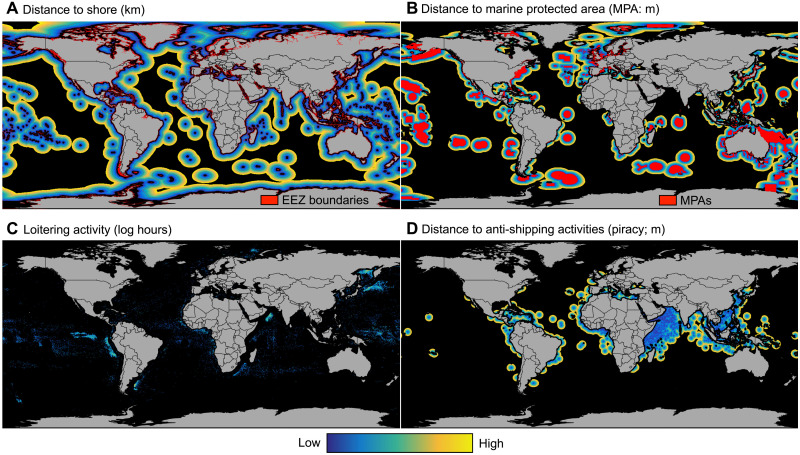
Potential behavioral drivers of suspected disabling events. Panels show distance to shore (**A**), distance to marine protected areas (**B**), loitering activity (**C**), and distance to anti-shipping activities (**D**). Distance drivers (A, B, and D) are clipped to 400 km to constrain models to proximal effects.

**Fig. 7.**
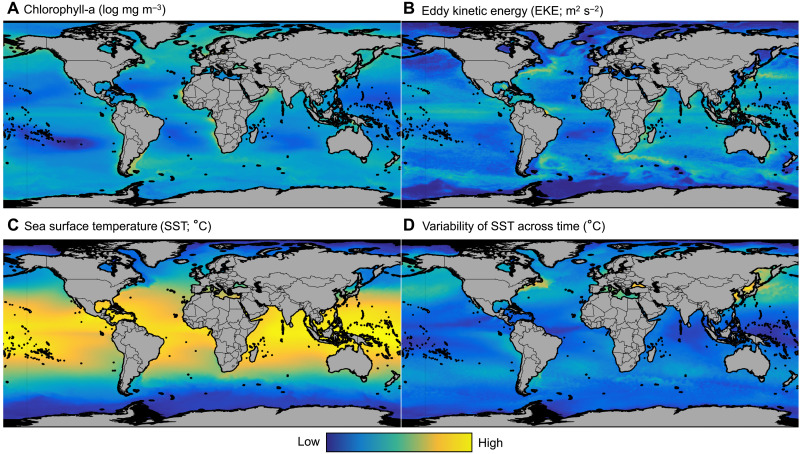
Potential environmental drivers of suspected disabling events. Panels show chlorophyll (**A**), eddy kinetic engergy (**B**), sea surface temperature (**C**), and the variability of sea surface temperature across time (**D**). Drivers are average conditions across the 2017-2019 time series.

To understand why vessels intentionally disable their AIS devices in certain locations and not others, we used boosted regression tree models to compare the relative importance of eight potential drivers of suspected disabling (see section S6). We selected four behavioral drivers: distance to shore (a proxy for distance to EEZ); loitering by transshipment vessels (a proxy for potential transshipment events); distance to reported antishipping activities (hereafter piracy), which are mainly reported pirate attacks; and distance to MPAs (Fig. 6) (*39*). While MPAs differ in which activities they exclude, we chose to include all MPAs after preliminary analysis, which revealed no relationship between disabling and no-take areas. We also selected four environmental drivers commonly used as proxies for the quality of fishing grounds (*40*): chlorophyll-a concentration, eddy kinetic energy, sea surface temperature, and the variability of sea surface temperature across time (Fig. 7). Using these eight drivers, we built individual boosted regression tree models for the four dominant gear types (squid jiggers, trawlers, tuna purse seines, and drifting longlines) and a full model that included all suspected disabling events (i.e., the four gear types listed above and additional gears such as gillnet and troll). Presences were grid cells in which there was at least one disabling event from 2017 to 2019. Absences were generated by randomly subsampling grid cells with fishing activity and no suspected disabling events across 2017–2019 to achieve a 1:1 ratio of presences to absences (see section S7.1).

Model performance was evaluated using three metrics: explained deviance, AUC, and true skill statistic (TSS). Explained deviance was calculated for each of the final models. AUC and TSS were calculated using 50 iterations of 75/25 cross-validation to explore model performance on novel data. For each iteration and each suspected disabling model, a new set of absences was randomly selected (while maintaining the 1:1 ratio of presences to absences). Then, new models were trained using a random 75% subset of the data and tested against the remaining 25% of the data.

## Supplementary Materials

### This PDF file includes:

Supplementary Materials and Methods Figs. S1 to S24 Tables S1 to S6 References
