# Supplementary material for: Hot spots of unseen fishing vessels
Source: Sci Adv. 2022 Nov 2;8(44):eabq2109. doi: 10.1126/sciadv.abq2109 (PMC9629714; doi:10.1126/sciadv.abq2109)
Supplement: Supplementary file 1 — Supplementary Materials and Methods Figs. S1 to S24 Tables S1 to S6 References [file sciadv.abq2109_sm.pdf]

Science Advances
AAAS

Supplementary Materials for

Hot spots of unseen fishing vessels

Heather Welch *et al.*

Corresponding author: Heather Welch, heather.welch@noaa.gov

Sci. Adv. **8**, eabq2109 (2022)
DOI: 10.1126/sciadv.abq2109

This PDF file includes:

Supplementary Materials and Methods
Figs. S1 to S24
Tables S1 to S6
References

Materials and Methods

1. AIS datasets

In 2017, approximately 60,000 active fishing vessels broadcasted their position over Automatic Identification System (AIS), representing roughly two percent of the world's 2.8 million fishing vessels. This AIS fleet is biased toward large vessels, covering the majority (52-85%) of vessels over 24m in length, while considerably fewer vessels (14-19%) between 12-24m and hardly any (<0.4%) vessels less than 12m (20). The high coverage of larger vessels is primarily the result of flag states adopting AIS measures stricter than the Convention for the Safety of Life at Sea (SOLAS), the international regulation governing AIS, which explicitly exempts fishing vessels. As a result, the AIS fleet is also biased toward vessels from upper/middle income countries and distant water fleets, as vessels fishing far from shore tend to be larger.

We acquired over 28 billion AIS messages from Global Fishing Watch (GFW) from 2017-2019. This time range was selected because, starting in 2017, the GFW AIS database contains data from both the Spire and Orbcomm AIS providers, providing a more complete dataset on AIS usage, and because model training and validation relies on a third party subset of AIS data provided by exactEarth for 2017-2019. Each AIS message consists of a timestamp, speed, course, location, and information on vessel identity such as Maritime Mobile Service Identity (MMSI) number and flag state. These data were processed using two convolutional neural networks to identify vessel characteristics and type of fishing activity, such as vessel length, tonnage, fishing gear type, and gear deployment events (for full details on these neural networks see (*7*)). These data were filtered to fishing vessels, as well as carrier vessels involved in transshipment, and further cleaned to remove highly inactive vessels (vessels with less than five days of AIS activity and less than one day of fishing in a year). Vessels that were offsetting their location or where multiple vessels were using a single MMSI number were also removed, which eliminated a few hundred vessels from the analysis. These vessels were identified as described in (*20*).

While we include all fishing vessels in our analysis, we focus on four classes of fishing vessels responsible for a large majority of suspected disabling events - trawlers, drifting longlines, tuna purse seines, and squid jiggers. Trawlers are defined as vessels that fish by towing a net through the water and include both bottom and mid-water trawlers. Drifting longline vessels deploy longlines attached to buoys that drift while tuna purse seines are defined by GFW as large purse seine vessels primarily fishing for tuna. Lastly, squid jiggers include vessels that fish while stationary using lights to target squid and other species, notably Pacific saury. The analysis also considers proximity of disabling events to loitering behavior by refrigerated cargo vessels capable of accepting transshipments of catch at sea.

The data were summed across the time-series, aggregated into quarter degree rasters (the coarsest resolution of the environmental datasets) and summarized using three fields related to fleet behavior: vessel days (Fig. S1A), fishing days (Fig. S1B), and suspected AIS disabling events (Fig. S2). Vessel days were calculated as the sum of days across the time-series that each fishing vessel of a fleet was present within a grid cell. Fishing days was the sum of days across the time-series that each fishing vessel of a fleet spent actively fishing within each grid cell. These data were also processed into suspected AIS disabling events – track segments with missing AIS data where we determined the AIS device was likely to have been intentionally disabled (Section 3). The analyses below were conducted using R version 4.0.4. statistical computing (*41*) and Python 3.7 (*42)*.

1.1 50 nautical mile restriction

AIS messages are recorded by terrestrial (T-AIS) and satellite (S-AIS) receivers. However, these receiver types differ in the range and consistency with which they can receive AIS messages. AIS was designed for line of sight communications, i.e., for communicating with nearby vessels within a few tens of kilometers. AIS devices continuously synchronize with nearby devices to organize their messages into one of 2,250 time slots, ensuring that all messages are received by all AIS devices within range. In this respect, T-AIS receivers will perform like AIS devices on vessels, receiving all messages from vessels within range, which is influenced by the elevation of the T-AIS antennae. In the GFW data, 99% of AIS messages recorded by T-AIS receivers come from vessels within 50 nautical miles of shore. Similarly, MarineTraffic reports the majority of their T-AIS receivers fully cover a range of 40 nautical miles. In contrast, satellites can receive messages from vessels within a few thousands of kilometers, an area far too large for all AIS devices to be synchronized. In areas of high vessel density, these messages can interfere with each other, causing the satellite to receive fewer AIS messages from each vessel. As a result, in areas with more vessels (usually near the coasts in highly developed regions) the number of AIS messages received for a vessel will generally decline as the vessel gets closer to shore before coming within range of a terrestrial receiver. Conversely, a vessel heading out to sea may see its AIS message count drop dramatically once out of range of terrestrial receivers. The effect of this steep gradient between terrestrial and satellite reception is to create long gaps in a vessel's AIS data. Therefore, we restrict our analysis to AIS gaps that start and end in waters more than 50 nautical miles from shore in order to eliminate these reception gaps.

2. Satellite AIS reception quality

2.1 Observed satellite AIS reception quality

Due to its design as a collision avoidance system, vessels broadcast AIS messages more frequently at higher speeds. To calculate the observed satellite AIS reception quality in an area - measured as the average number of AIS messages received by satellites per vessel per day - we first filtered the AIS dataset to only consider positions with speeds greater than two knots for Class B and speeds under 14 knots while not at anchor for Class A. At these speeds, Class B broadcasts once every 30 seconds and Class A usually broadcasts once every 10 seconds. Below two knots, Class B devices broadcast every three minutes and above 14 knots Class A devices transmit more frequently than once every 10 seconds. Fishing vessels rarely travel faster than 14 knots, and therefore we did not lose many AIS positions due to this cutoff. Imposing these speed filters is important because counts of AIS messages are influenced by how vessels commonly operate in an area (e.g. high speed steaming in shipping lanes, loitering near ports). The majority of AIS messages are from non-fishing vessels, and thus these vessels will dominate reception calculations (*7*). Building a reception map that included vessels broadcasting outside these bounds would thus create an overly pessimistic reception map for Class B fishing vessels and an optimistic reception map for Class A fishing vessels, as many cargo vessels in shipping lanes travel faster than this speed and thus broadcast more frequently.

Next, to account for areas where no AIS positions were received, we linearly interpolated the hourly position of every vessel. GFW uses a segmentation algorithm when processing AIS data to assign positions to logical tracks by MMSI (*7*). The algorithm assigns subsequent AIS positions to a new track segment after a 24 hour gap in AIS messages, and hourly interpolation was not performed between track segments. We chose not to remove AIS disabling events prior to inferring AIS reception for multiple reasons. First, we felt estimates of satellite reception were a prerequisite for identifying suspected disabling events and expected that the number of AIS gaps far exceeded the number of actual disabling events. Indeed, while suspected disabling events are ~10% of all AIS gaps by fishing vessels in the study area (>50 nm from shore), they are just one percent of all fishing vessel gaps greater than 12 hours in the AIS data. Second, due to challenges with terrestrial and satellite AIS reception nearshore, this analysis only focuses on identifying suspected disabling events more than 50 nm from shore. Thus, only suspected disabling events >50 nm offshore could be removed prior to inferring reception quality, which would create discrepancies in our estimates of nearshore (< 50 nm) versus offshore (> 50 nm) reception.

The observed reception quality was then calculated separately for Class A and Class B as the average number of AIS positions received by vessels at one degree resolution (Fig. S3). For both Class A and B devices, S-AIS reception quality decreases close to shore in many areas due signal interference (see Section 1.1). Importantly, Class A AIS devices have a higher output power than Class B (12.5 versus 2 watts), which also increases the likelihood Class A messages will be received by satellites. The calculation and subsequent modeling of reception quality was

performed for each month in the dataset (2017-2019) to account for temporal changes in reception as more AIS satellites came online.

2.2 Predicted reception quality

Vessel presence in the ocean is highly concentrated in some areas but very sparse in others, and the empirical estimates of reception quality in a given one-degree cell are sensitive to the amount of vessel activity in that cell. Therefore, we chose to smooth observed reception quality by interpolation with a radial basis function (RBF). A RBF was chosen because it provides a relatively straightforward way to interpolate large datasets on an unstructured, global mesh and is well behaved in areas with low point density (*43*). This interpolation was performed using the scipy Python module (scipy.interpolate.Rbf) with a multiquadric radial basis function and a smoothing parameter equal to one. To minimize the influence of noise, cells with a very low amount of activity (< 15 hours) in a month were down-weighted by using the difference in activity from the minimum 15 hour threshold as a third dimension in the interpolation, effectively placing low activity cells further from the interpolation surface. Figure S4 shows the predicted AIS reception quality for Class A and Class B, respectively, across the full study period. We use the predicted AIS reception quality to set a minimum threshold of ten positions per day in order to exclude AIS gap events from areas of extremely unreliable AIS reception.

The residuals (observed minus predicted reception) for both Class A and Class B models are normally distributed around zero (Fig. S5). When examining the spatial distribution of residuals, we restricted the analysis to cells with at least 24 hours of activity and capped reception quality at 100 positions per vessel day. These restrictions allowed us to better understand the interpolation's performance in areas where reception is of most concern (Fig. S6). This examination suggests under-prediction of reception for Class A AIS primarily occurs in areas of low reception and is evenly distributed in these areas. Over-prediction of reception is concentrated in several regions, including near the Faroe Islands, the Northern Indian Ocean, and Indonesia. Residuals for Class B AIS are much more spatially heterogeneous (Fig. S6). Areas of over-prediction for Class B reception quality include the North Pacific, the Gulf of Guinea, Western Australia, and in the high seas around the Galapagos. Notable areas where Class B reception quality is under-predicted include coastal West Africa, the Western Indian Ocean, and the Southern Atlantic near Argentina. However, reception quality in all of these areas is still above the ten positions per day threshold. Importantly, over/under-prediction of reception quality only affects our results when the prediction moves the reception quality in a cell above or below this threshold. A small number (0.8%) of suspected disabling events representing less than 0.5% of the total time lost to disabling occurred in areas where reception quality was over-predicted above the ten position threshold. Conversely, an additional 176 events were omitted due to the under-prediction of reception quality below the ten position threshold. If included, these events would only increase the total time lost to disabling by approximately 0.1%.

2.3. Restricting to gaps longer than 12 hours

Because AIS messages are not broadcast at a constant rate and satellite reception influences how frequently AIS messages are received, analyzing AIS disabling first requires defining the minimum period between AIS messages that is considered a gap. We restricted our analysis to only include gaps longer than 12 hours because satellite reception can vary quite substantially at time scales shorter than 12 hours. There are several dozen satellites between Spire and ORCOMM, and they orbit the earth roughly every 110 minutes. A satellite can usually receive messages from any vessel within the satellite's field of view, which is usually between 2000 and 3000 km depending on the altitude of the satellite. The orbits are such that even in high reception areas, there can be a few hours without many satellites within range, and thus satellite reception varies quite significantly at short time ranages. However, when averaged over 12 hours or longer, satellite reception is roughly constant.

To illustrate how reception is variable at times shorter than 12 hours, we calculated the location and altitude of every Spire and ORBCOMM satellite for every second for two months. We then calculated, for every minute of the day, the average number of satellites that could be seen at a few given locations on earth. That is, how many satellites were over the horizon, and thus capable of receiving a signal from a vessel. While the patterns are different at different latitudes (Fig. S7 A,D,G,J), a Fourier analysis shows that at all latitudes except very high latitudes, the 12 hour period has the strongest signal and reception quality is therefore roughly constant after 12 hours (Fig. S7 B,E,H,K). Further, we calculated the standard deviation of the number of satellites overhead for different time windows, and found that the standard deviation dropped quickly until 12 hours, after which it remained low (Fig. S7 C,F,I,L).

3. Detecting suspected AIS disabling

All gaps in AIS transmission at least 12 hours in length were identified (section 2.3). Poor satellite reception (Section 1.1) and low broadcast rates (Section 2.1) may cause AIS transmission gaps that do not indicate intentional disabling by vessels. Therefore, a classification model was developed to identify AIS transmission gaps that are most likely due to intentional disabling as a function of expected satellite reception quality and AIS broadcast rates.

3.1 Rule-based classification model specification

Four features were calculated for each AIS transmission gap, including 1) predicted monthly reception quality where the gap started (described in Section 2.2); 2) number of positions within 12 hours prior to the gap; 3) number of positions within 18 hours prior to the gap; and 4) number of positions within 24 hours prior to the gap. Metrics were limited to when AIS transmission

ceases and the gap begins so that this model could be operationalized to identify suspected disabling events in real or near real time without relying on metrics related to when AIS transmissions begin again post gap events. Reception quality (feature 1) is based on the average transmission frequency of all vessels operating in an area, whereas individual vessel position ping rates (features 2-4) account for variability in the transmission frequency of individual AIS devices. Since a vessel can be in a high reception area but ping infrequently and vice versa, this set of features allowed us to test the classification power of both internal and external factors. A set of rule-based classification models that apply thresholds to combinations of these features in an IF-THEN framework were developed, including models using only reception, models using only individual vessel position ping rates, and models that combine both types of features (Table S1).

Repeated k-Fold Cross Validation was used to identify the ping rates (features 2-4) and/or reception (feature 1) thresholds with the highest performance for identifying intentional disabling events. Since the objective of this study is to identify suspicious and potentially illegal behavior, we wanted to prioritize precision to limit the potential for false positives while still accounting for overall accuracy and recall. Therefore, model performance is calculated with F0.5 score, a variant of an F1 score that weights precision twice as much as recall by setting β=0.5, as described in (*44*). Models were evaluated for every whole integer for individual vessel position ping rate threshold, k, where $1 <= k <= 60$ positions in the specified time period (12 or t hours before or after gap), and reception threshold, j, where $11 <= j <= 60$ (Fig. S8). The lower bound of j was set at eleven as gaps that start in areas with a reception of ten or less are already excluded from the gaps dataset (Section 2.2).

3.2 Labeled gaps

To better assess whether AIS transmission gaps in the GFW AIS dataset were indeed true (i.e. intentional) AIS disabling events, we acquired AIS data from a third AIS provider, Exact Earth, for a subset of vessels in 2017 to 2019. The subset included 4,403 unique MMSI from 89 flag states that collectively covered 112,567 transmission gaps in the GFW AIS data for 2017 to 2019 that are at least 12 hours long, start in areas with a reception greater than ten positions per vessel day, and start at least 50 nautical miles from shore. This subset of gaps to be labeled was investigated and compared to the full set of gaps and was found to be reasonably representative both spatially and across gear types (Figs. S9 and S10).

We then merged the exactEarth AIS data with the GFW AIS data to create a labeled dataset consisting of transmission gaps labeled as “true” gaps - those gaps common to both datasets and thus suspected of occurring from intentional disabling - and “false” gaps - those which are unlikely to be due to intentional disabling and instead were likely caused by technical issues. Gaps were labeled as “true” if there was still a gap of at least 12 hours after adding in the Exact

Earth positions. If the gap was not longer at least 12 hours after doing this, then it was labeled as “false”. This criteria was set based on the patterns of satellite coverage outlined in Section 2.3. The final labeled data set was divided into separate training and test sets using a 70-30 group shuffle split before performing model selection. The data was grouped on MMSI so that all of the gaps from a given MMSI were either entirely in the training or the validation set. Having gaps from the same MMSI in both sets was found to cause instability in the cross validation scores. Due to the grouping, the final split was actually 72-28 but will be referred to as the 70% training set and 30% test set for simplicity.

3.3 Model selection

Model selection was performed using Repeated k-Fold Cross Validation on the 70% training set using the standard of ten folds repeated ten times. Model performance was evaluated using the F0.5 score on the validation set for each cross validation run. This is separate from our use of F0.5 score during model fitting as described in Section 3.1, but it is done for the same reason of wanting to prioritize precision over recall. The average of the F0.5 scores for these 100 runs was calculated for each potential model to find the optimal model (Table S2). Once a final model was selected, it was trained on the full 70% training set and accuracy metrics were calculated using the independent 30% test set.

3.4 Model selection results

The distribution of the F0.5 scores for each model can be found in Figure S11. The model with the optimal score was *rec_12hb* (F0.5=0.741). However, we selected the *12hb* model for gaps classification, which scored nominally lower (F0.5=0.739). Because the *12hb* model does not rely on satellite reception, it is easier to operationalize in order to eventually produce a suspected disabling dataset that updates in real-time and promotes applied usage of the data. The *12hb* model was then parameterized by training on the full 70% training set and was determined to have an optimal vessel position ping rate threshold, k, of 14. This means that gaps with at least 14 positions in the 12 hours before the gap start will be considered to be suspected disabling gaps. This model was used to generate the set of gaps (hereafter: suspected disabling events) used in the remainder of the analysis.

Using the 30% test set withheld before cross validation, this model was shown to work well to limit false positives with a false positive rate of 3.72% and a precision of 0.86 (Fig. S12). The F0.5 score on the test set is 0.718 and the accuracy is 66% with accuracy loss due largely to false negatives (42.93%). This is expected as model selection was aimed at being conservative and minimizing false positives.

The optimal parameterization for each model demonstrated that the addition of individual vessel position ping rate features greatly improved upon the performance of the reception-only model (Fig. S8). However, the addition of reception to position features showed negligible changes in performance from those with position features only (Table S2). Evaluating model performance at each reception threshold showed a mostly stable or nominal increase in optimal model performance with increasing reception followed by a steep decline around 35 positions per vessel day (Fig. S13). These results indicate that using a reception threshold higher than the lower limit of 10 positions per vessel day is not beneficial to model performance. This phenomenon makes sense when considering the use of F0.5 instead of F1 score and that individual vessels within areas of the same reception quality can have vastly different position ping rates. Even in the lowest reception areas, a subset of vessels can still have a sufficient number of positions surrounding their gaps to strongly indicate that the sudden absence of positions is due to intentional disabling. Increasing the reception quality threshold falsely classified these true gaps as false, leading to a decrease in model performance.

4. Suspected AIS disabling dataset

The final dataset of 55,368 suspected disabling events from 2017-2019 was created by applying the chosen model to all AIS transmission gaps of at least 12 hours that start in areas more than 50 nautical miles from shore and with a reception greater than ten positions per vessel day. These gap events represent those AIS transmission gaps for which we have the most confidence that vessels intentionally disabled their AIS devices. This final dataset of 55,368 suspected disabling events included events by 5,269 distinct MMSI from 101 flag states, with a median duration of 23.5 hours. The median distance traveled during suspected disabling events was 78.6 kilometers. However, the distributions of duration and distance traveled were both heavily right-skewed (Fig. S14). China, Taiwan, Spain, and the United States were the top four flag states in terms of suspected disabling events, collectively accounting for more than 65% of all suspected disabling events (Table 1). With regard to vessel class, drifting longlines, squid jiggers, trawlers, and tuna purse seines accounted for over 92% of suspected disabling events (Table 1).

5. Spatial allocation of disabling events and time lost to these events

To quantify the scale of the management problem presented by AIS disabling, we developed methods to approximate where vessels operate when their AIS is disabled, thus allowing us to spatially allocate the time vessels spend with their AIS disabled. This analysis required 1) determining if the vessels were, in fact, at sea (and not at port) when their AIS was disabled, and 2) for vessels at sea, estimating where they likely traveled between where they disabled their AIS and turned it back on. This analysis was done using GFW AIS data and not the merged dataset

described in section 3.2. Global Fishing Watch processes its AIS data daily with an automated set of algorithms which generate several features important for this analysis and integrating the exactEarth subset into this process was not feasible.

To identify if vessels were still at sea, we analyzed the median and mean voyage time of vessels on high seas voyages to determine if they likely visited port in the interim. To spatially allocate time lost to disabling events, we explored two different methods of varying complexity (linear interpolation and rasterized probabilities) to estimate where vessels operated with their AIS disabled. For our key findings (total and fraction of fishing vessel activity obscured by disabling events, and disabling hotspots; Figures 1&2, Table 1) the two methods provided very similar results (Table S3). Thus our main results use the simplest of these methods (linear interpolation), but both are described and reported here.

5.1 Time at port versus sea

The majority of disabling events are short in duration, with 94% being shorter than two weeks. Longer events, though, may account for disproportionately more activity, simply because each event has more time. A key question is if a vessel is at sea or port when its AIS is disabled. Because vessels often turn off their AIS in port, a long disabling event may be a sign that the vessel has gone to port and is not at sea. On the other hand, some vessels can spend many months or longer than a year at sea (*15*).

We analyzed GFW's database on the length of vessel voyages from 2017 to 2019, and found that the mean time between port visits for voyages farther than 50 nautical miles from shore was 27 days, and voyages longer than 73 days accounted for half of the total vessel activity. If a disabling event is longer than these values, it is more likely that a vessel spent some of the time during a disabling event at port. Thus, for calculating the likely lower bound on the time lost to disabling events, we only consider disabling events significantly shorter than the mean voyage time. We chose a time of 14 days, about 30 percent below the mean voyage. Because voyages are mostly longer than 14 days, our data supports that the vast majority of the time in these disabling events was spent at sea and not at port. However, it is possible that the longer events (> 14 days) represent mostly time at sea and not at port. Therefore, our upper bound on time lost in the high seas allocates all time in disabling events of any length.

5.2 Spatial allocation of disabling events

Time lost to suspected AIS disabling is equal to the total duration (hours) of AIS disabling events and is spatially allocated based on two different methods. We spatially allocate activity in disabling events at one degree (grid size of ~111km at the equator).

In the first method (linear interpolation), we interpolate positions of vessels between the start and end of the disabling events positions, assuming that the vessels travel in a straight line when their AIS is off. While linear transits are likely not the case, when using a relatively low-resolution grid (one degree), it provides an estimate of where vessel activity took place during disabling events.

In the second method (rasterized probabilities), we used broadcasting vessels to build probabilistic rasters of where a vessel likely traveled between two positions that are a given distance and time apart. For instance, to determine where a drifting longline may have gone in a disabling event that was three days in duration and 50km in distance, we compiled all tracks in the AIS dataset where a drifting longline had two positions 50km and three days apart and that vessel left its AIS on for the three days in between. Using these tracks, we built a probabilistic raster showing the positions these vessels visited in the interim. The result is a heatmap of where vessels with that starting and ending point were likely to visit.

Because each vessel class has distinctive movements, we built probabilistic rasters for each of our five vessel classes (drifting longlines, trawlers, squid jiggers, tuna purse seines, and other). We identified activity at distances of 0, 10, 20, 40, 80, 160, 320, 640 and 1280 km, and durations of 0.5, 1, 2, 4, 6, 8, 10, 12, 14, 16, 18, 20, 25, 30 and 35 days. These distances and times were chosen to make sure almost every disabling event had a raster that was relatively close in duration or distance. All combinations of duration and distance were paired, with a different raster for each vessel type (675 of cases in total). Each of these cases, except for some that were physically impossible (e.g. 1280 km in 12 hours) had several hundred examples of vessels to draw on. We then plotted every single position that a vessel visited between the start and end point, gridded the amount of time spent in each grid cell (these probability rasters were gridded a resolution of 10km, 10x finer than the resolution of our final analysis), and then averaged these values across all vessels in a given vessel class (Figure S15 shows some of these probabilistic rasters). For each disabling event, we selected the probability raster that best corresponded in distance, duration, and gear type to the disabling event in question. We then apply a similarity transformation to the probability raster so that its start and and end points correspond to those of the disabling event. The time spent in each grid cell is also adjusted so that the entire raster time sums to the duration of the disabling event. Figure S16 shows an example of a few disabling events spatially allocated using this method and using the interpolation method.

When using a one degree grid, these two methods (linear interpolation and rasterized probabilities) yield very similar results, and our key findings are essentially identical at short time periods (< 2 weeks) but results deviate at longer time periods (Table S3). For disabling events under one week (84% of the events), almost all activity is within one degree (Fig. S17). For events between one and two weeks, about 80% is within one degree. This percentage drops with longer disabling events, and for events four weeks and longer (~3% of events over 4

weeks), only about half of the time lost is close to a line between the two locations (Fig. S17). Figure S18 shows the spatial allocation of time in disabling events for both the rasterized probability and linear interpolation for disabling events shorter than two weeks and for all disabling events.

5.3 Total time and fraction of time lost to disabling events

To estimate the time lost to AIS disabling, we used both the linear interpolation and rasterized probabilities methods to spatially allocate disabling events, and summed the total time spent more than 50 nautical miles from shore and above a minimum reception quality of ten positions per day. The linear interpolation method showed slightly higher time lost to disabling (~5% more time) compared to the rasterized probability method (Table S3). This is because the rasterized probability method smooths out interim disabling event positions, placing some positions (and therefore some of the time lost) within 50 nm from shore and outside of our study area (e.g. Fig. S16). Nonetheless, the numbers differ very little, as shown by Table S3. The lower bound on time lost only considers disabling events under two weeks. the upper bound includes all disabling events, as described in Section 5.1.

The fraction of time lost to disabling events was calculated as the time lost to disabling events divided by the time at sea for all fishing vessels in the study area. The time at sea is the total time broadcasting including all gaps in transmission (which includes both intentional disabling and gaps due to poor AIS reception). Using either method for allocating these longer gaps (raster method or interpolation), we estimate that 3-6% of fishing vessel activity is lost to suspected AIS disabling events, although there are slight differences for some of the gear types between the two methods (Tables 1 & S3).

To spatially allocate the fraction of time lost to disabling events (Figures 1&2) we only include disabling events shorter than two weeks in duration. We have the most confidence in our ability to map these events because our analyses demonstrate that under two weeks 1) vessels are unlikely to have visited port (Section 5.1), 2) there is high agreement between the linear interpolation and rasterized probability methods (Section 5.2), and 3) 80% of vessels are within one degree of a straight line connecting the start and end of disabling events (Fig. S17).

6. Behavioral and environmental drivers

To understand why vessels intentionally disable their AIS devices, we acquired data on potential behavioral and environmental drivers from multiple sources (Table S4).

6.1 Behavioral drivers

GFW has compiled a dataset of refrigerated cargo vessels ("carriers") from which it has identified two-vessel encounters and single-vessel loitering behaviors using rule-based methods (*7*, *17*). Loitering behavior by carrier vessels is identical to behavior during two-vessel encounters with fishing vessels, except that the potential second vessel is not visible on AIS. Therefore, loitering behavior in an area may indicate activity by fishing vessels that have disabled their AIS. Loitering activity by carrier vessels was converted into a quarter degree raster showing the total number of loitering hours across 2017-2019 in each grid cell. We log transformed loitering hours to remove the left skew caused by low loitering hours in many grid cells.

We acquired a high resolution land shapefile from the Pacific Islands Ocean Observing System. To identify marine protected areas, we acquired the World Database on Protected Areas (protectedplanet.net) and selected all designated marine protected areas regardless of International Union for Conservation of Nature status. Anti-Shipping Activity Messages (hereafter: piracy) were downloaded from the US National Geospatial-Intelligence Agency across the full available time-series (February 1979 through December 2019). These are mainly reported pirate attacks (88.6%) but include other encounters such as kidnaping, naval engagement, and suspicious approaches. This long time-series was selected to account for the role of historically dangerous waters in driving vessels to disable their AIS devices.

Distances from shore, marine protected areas, and piracy events were calculated and resampled into quarter degree rasters (Fig. 6). All distance drivers were reclassified beyond 400 km to a constant value. In the case of distance from shore, the 400km threshold was measured from 200 nm from shore (the distance of most Exclusive Economic Zone boundaries). This threshold was selected to provide enough distance values to understand the statistical relationship between our response variable and predictor variables in our models that are described below, i.e. within 400 km there are roughly 16 quarter degree grid cells over which variation in the response variable can be calculated. The reclassification removed the ability of distances beyond 400 kilometers to explain any of the variation in the response variable. This was an important step because models might detect correlative relationships between suspicious disabling events and features that are very distant, for example across an ocean basin. While such a relationship may have statistical significance, it is unlikely to have geographic or behavioral meaning.

6.2 Environmental drivers

Data for environmental drivers (Fig. 7) were acquired from Copernicus Marine Environment Monitoring Service from 2017-2019 (Table S4). This time-series required us to use both reprocessed and near real-time products for chlorophyll-a and eddy kinetic energy. All data were acquired as daily rasters at quarter degree resolution. To produce sea surface temperature and chlorophyll-a fields, the daily rasters were averaged across 2017-2019 to produce a climatology for each product. Chlorophyll-a was $\log_{10}$ transformed. To calculate the variability of sea surface

temperature, the temporal standard deviation of the daily sea surface temperature in each grid cell was calculated across 2017-2019. The following formula was used to calculate daily eddy kinetic energy, where *i* is an individual grid cell, and *vgosa* and *ugosa* are the meridian and zonal components of geostrophic velocity anomalies, respectively:

Equation 1

$$Eddy\ Kinetic\ Energy_i = \frac{1}{2} * ({ugosa_i}^2 + {vgosa_i}^2)$$

Daily eddy kinetic energy rasters were then averaged across 2017-2019 to produce a climatological raster (Fig. 7).

7. Boosted regression tree models

The probability of suspected disabling events was modeled as a function of environmental and behavioral drivers using a popular machine learning method called boosted regression trees (BRTs) via the 'dismo' R package (*45*). BRTs can fit complex nonlinear relationships and are robust to a wide variety of data types and distributions, and have proven application for understanding the drivers of fishing fleet distributions (*40*, *46*). The BRT output included a measure of the relative importance of behavioral and environmental drivers for suspected disabling events and fishing activity (Fig. 3). Relative importance (or relative contribution) is a measure of each variable's influence on the response, and is based on the number of times each variable is selected for splitting across all trees (*45*). Importance is scaled across all variables such that the sum adds to 100, producing relative importance, where higher values indicate stronger contributions (*45)*.

We modeled the probability of suspected disabling using fishing activity as absences to understand if and how these two inherently related behaviors diverged. Prior to modeling, data for both behaviors were restricted to beyond 50 nm from shore to match the dimensions of the suspected disabling dataset. Five models were built for suspected disabling events: a full model containing all the data, and four individual gear type models for drifting longlines, trawlers, squid jiggers, and tuna purse seines. We built individual gear type models to understand how amounts of disabling vary between gear types and if the differing levels of management oversight across gear types affected fleet behavior. The full model contained all disabling events in the dataset, 63% of which were from the four gear types for which individual models were built (drifting longlines, trawlers, squid jiggers, and tuna purse seines). The remaining 37% of the data came from gear types without enough disabling events to build robust models (e.g. set gillnets, trollers), or from vessel tracks that could not be classified to the gear level by the GFW neural network.

7.1 Presences and absences

For our boosted regression tree modeling approach, we specified presences and absences from our dataset (Fig. S19A). For suspected disabling events, presences were grid cells in which there was at least one disabling event from 2017-19, ranging from 28874 presences in the full model to 2342 presences for tuna purse seines (Table S5). Absences were defined by randomly subsampling locations with fishing activity and no suspected disabling events across 2017-19. This method selected locations where suspected disabling could have occurred, but was not detected. For the full suspected disabling events model, absences were subsampled across all locations with fishing activity. In the individual gear models, absences were subsampled across locations with fishing activity from the target gear type (e.g. absences for trawlers were selected from grid cells in which trawlers fished but did not disable their AIS devices). For all models, a presence:absence ratio of 1:1 was used, following results that this ratio works best for BRT models (*47*).

7.2 Model fitting and validation

A binomial distribution was used for all BRT models, which is appropriate for presence/absence data. BRTs were built with a bag fraction of 0.6, and a tree complexity of three (*45*). The learning rate was varied across models between 0.1 and 0.001 depending on the number of data points to ensure at least 1,000 trees were fit for each model (*45*). The resultant BRTs provided two important outputs: the relative importance of drivers, which allowed us to compare the relative importance of environmental and behavioral drivers across models, and the partial responses to the drivers, which allowed us to understand how suspected disabling and fishing activity respond to the various drivers.

Model performance was evaluated using three metrics: explained deviance, Area Under the Receiver Operator Characteristic Curve (AUC), and True Skill Statistic (TSS). Explained deviance measures how well models can capture patterns in the response variable, and is a measure of model explanatory power. AUC and TSS are metrics that reveal the ability of models to accurately discriminate between presence and absences in novel training data, and are thus measures of model predictive skill. Explained deviance was calculated for each of the final models. AUC and TSS were calculated using 50 iterations of 75/25 cross-validation to explore model performance on novel data. For each iteration and each suspected disabling and fishing activity model (n = 10), a new set of absences was randomly selected (while maintaining the 1:1 ratio of presences to absences). Then, new models were trained using a random 75% subset of the data and tested against the remaining 25% of the data.

Model performance was high across the five suspected disabling models (Table S6). Explained deviance ranged from 12.17% in the drifting longline suspected disabling model to 61.42% in the trawler model. Predictive skill was high across the 5 models, with average AUC values of 0.83 and average TSS values of 0.53. Standard deviations were low, indicating that performances were robust across different model iterations.

As a point of comparison, we built full models and four gear-specific models for vessel presence and fishing activity following the methods above. Vessel presence, fishing activity, and suspected disabling are modeled as nested behaviors: vessel presences represent where vessels choose to go including behaviors such as traveling to fishing grounds and ports; fishing activity is a behavioral subset of vessel presence locations where vessels choose to fish, and suspected disabling is a subset of fishing activity locations where vessels chose to disable their AIS devices. For the vessel presence models (Fig. S19C), presences were grid cells with at least one hour of vessel presence across the 2017-19 time-series, and absences were generated by randomly sampling locations with no vessel presence across the time-series (i.e. background sampling). For fishing activity (Fig. S19B), presences were grid cells that had been actively fished for at least one hour across the time-series, and pseudo-absences were generated by randomly sampling locations with vessel presence and no fishing activity across the time-series. For suspected disabling (Fig. S19A), presences were grid cells with at least one disabling event across the time-series, and absences were generated by randomly sampling locations with fishing activity and no disabling events across the time-series (same models as described above in Section 7.1). As such, these three sets of models test three different hypotheses:

1. Are locations with vessel presence different from the background environment?
2. Are locations with fishing activity different from locations with vessel presence?
3. Are locations with suspected disabling different from locations with fishing activity?

We explored the niche separation of each behavior by comparing the marginal effects of the most important variable in each disabling model to the corresponding marginal effects in the fishing activity and vessel presence models (Fig. S20). In the full model, the marginal effect of loitering activity plateaued at 100 hours for suspected disabling versus ~10 hours for fishing activity and vessel presence. For drifting longlines the marginal effect of distance to shore showed a peak around 200 nm for suspected disabling, while fishing activity and vessel presence were low inshore of 200 nm, increased steeply, and then remained offshore of 200 nm. For tuna purse seiners, the marginal effect of SST rose steeply between 20 and 30 °C for vessel presence, while the marginal effect for fishing activity and suspected disabling did not show a strong response. For squid jiggers, the marginal effect for loitering had a distinct peak between 100-1000 hours that was not present for the other two behaviors. For trawlers, the marginal effect of chl-a indicated a higher occurrence of suspected disabling in productive waters, while fishing activity and vessel presence were more broadly distributed.

8. Squid jigger disabling and loitering

Loitering activity was the most important predictor in the squid jigger model for suspected disabling, with the occurrence of suspected disabling increasing as loitering activity increased (Fig. 3 and 4). To understand why loitering was more important in the suspected disabling model compared to the fishing activity model, we examined the proximity of suspected disabling events, fishing activity, and vessel presence to loitering events.

To calculate relative proximity to loitering events, we interpolated all fishing activity, vessel activity, loitering activity, and disabling events to a constant temporal grid, so we had the likely location of fishing, vessels, loitering, and disabling events at every hour. For each fishing and vessel position and each disabling event, for each hour, we then calculated the distance to the closest loitering event. We then calculated the fraction of fishing activity, vessel activity, and disabling events that were within a given distance from loitering events. We found that squid jigger suspected AIS disabling events occur considerably closer to loitering events than fishing activity or vessel activity (Fig. S21).

It is important to note that, due to the high variability of satellite reception quality at time scales shorter than 12 hours (Section 2.3), we only consider suspected disabling events longer than 12 hours in duration. Transshipment events generally take less than 12 hours, and thus we are likely underestimating the importance of loitering events in the BRT models.

9. Suspected disabling events adjacent to EEZ boundaries

We compared the ratio of suspected disabling and fishing activity within 100 km from EEZ boundaries for each EEZ and flag state to investigate if there is more suspected disabling than would be expected by the amount of fishing activity (Fig. S22). To assess the pairwise relationships between flag states and EEZs involved in EEZ-adjacent disabling, suspected disabling events within 100 kilometers of an EEZ were associated with the nearest EEZ. For this analysis, version 11 of EEZ boundaries was downloaded from marinergions.org. These data were summarized as the total number of suspected disabling events between each EEZ and flag state pair, e.g. the total number of suspected disabling events in Spanish fleets adjacent to the Liberian EEZ. As a point of comparison, we also assessed the pairwise relationships between flag states and EEZs involved in EEZ-adjacent fishing. All grid cells that were actively fished within 100 kilometers of an EEZ were associated with the nearest EEZ. These data were summarized as the total number of fishing days between each EEZ and flag state pair, e.g. the total number of days Spanish fleets fished in waters adjacent to the Liberian EEZ. For each EEZ-flag state pair, the number of suspected disabling events and fishing days were converted into the percentage of total EEZ-adjacent suspected disabling events and fishing days, respectively. This allowed us to

identify EEZs and flag states that had higher percentages of total suspected disabling than expected based on their total fishing days.

In addition, we explored the relative percentages of suspected disabling and fishing activity within or adjacent to EEZs with overlapping claims. The EEZ shapefile contains an attribute for polygon type (“200NM”, “Joint regime”, “Overlapping claim”). Disabling events and fishing activity that occurred within 100 kilometers from EEZs were associated with the nearest EEZ, and the percentage of these events within or adjacent to EEZs with overlapping claims was calculated. Four percent of disabling events and 2% of fishing activity was within or adjacent to EEZs with overlapping claims, which comprise 11% of EEZ polygons. A two proportion Z-test indicated that these percentages were significantly different ($p\text{-value} < 0.0001$). EEZs with overlapping claims that had the most disabling events were the Chagos Archipelago (claimed by the UK and Mauritius), the Falkland/Malvinas Islands (claimed by the UK and Argentina), and the Kuril Islands (claimed by Japan and Russia).

Supplementary Figures

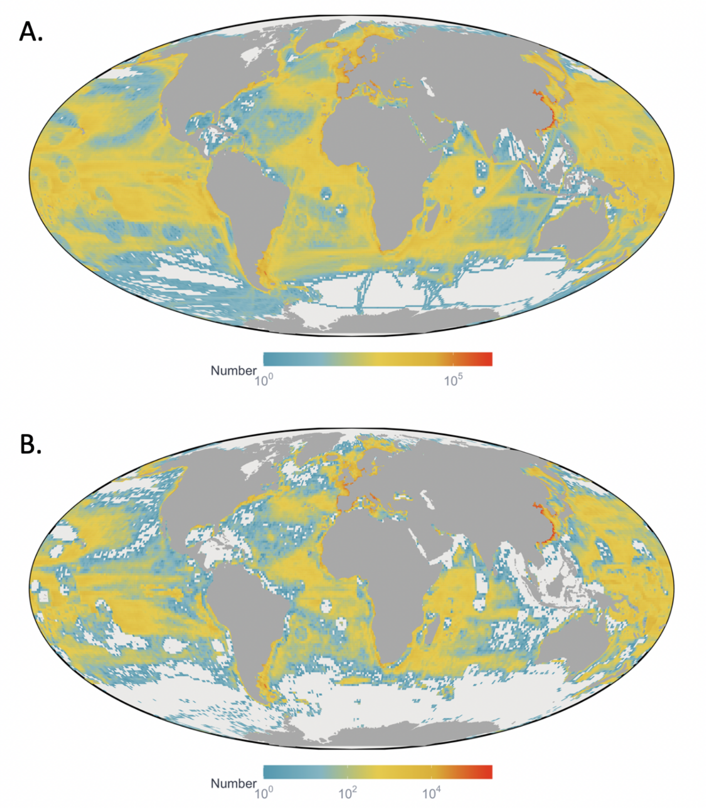

Figure S1. The Global Fishing Watch AIS dataset: vessel days (A) and fishing days (B).

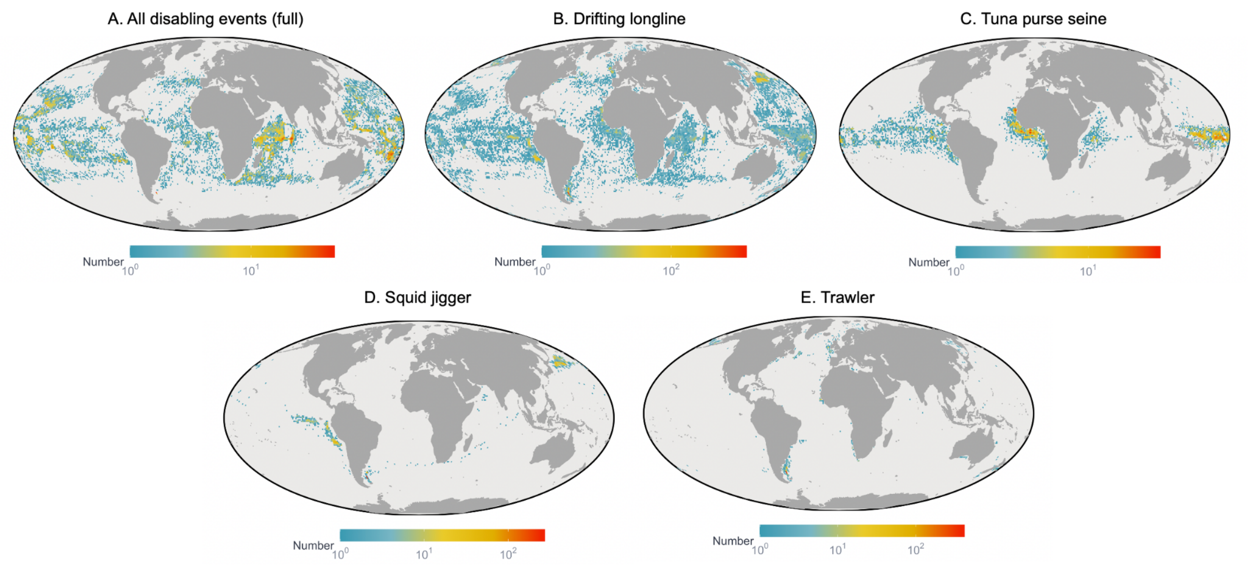

Figure S2. Number of disabling events across 2017-19 for the full dataset (A), drifting longines (B), tuna purse seines (C), squid jiggers (D), and trawlers (E). Disabling events are aggregated to 1° to increase visibility.

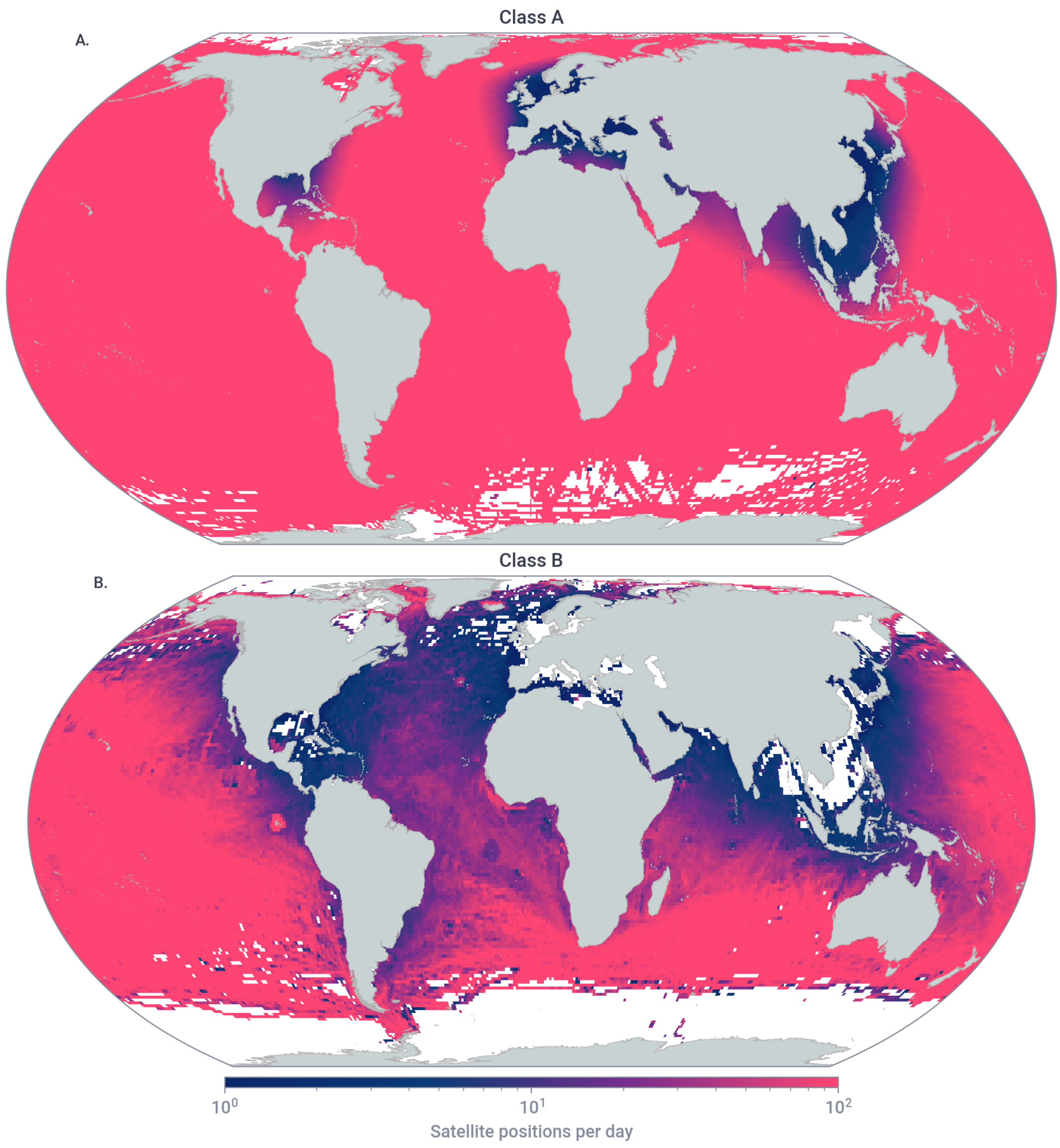

Figure S3: Observed reception quality in units of AIS positions ("pings") per vessel day for Class A AIS (A) and Class B AIS (B). White areas represent cells with no observed data in the study period.

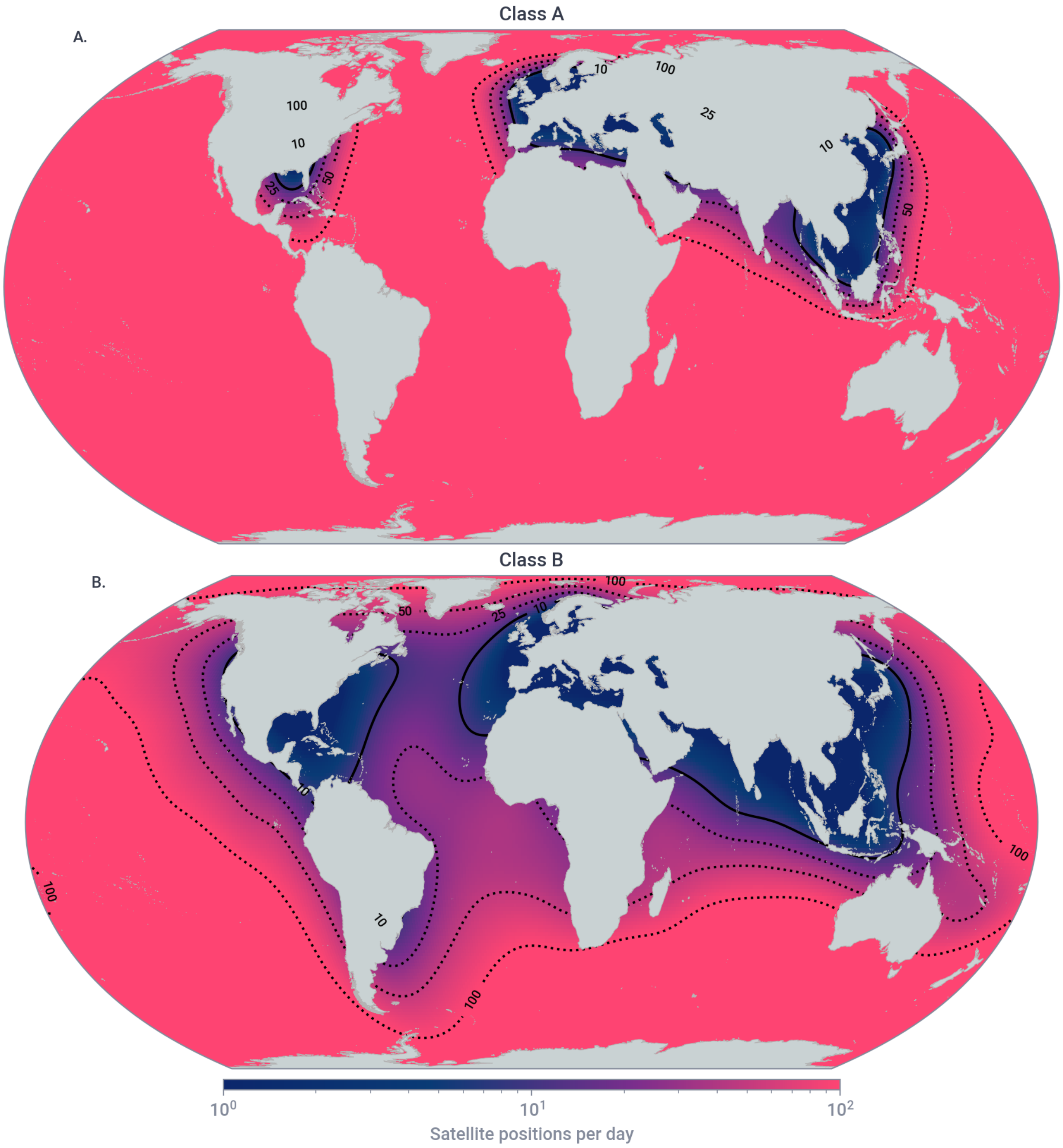

Figure S4: Predicted reception quality in units of AIS positions ("pings") per vessel day for Class A AIS (A) and Class B AIS (B). Contour lines are drawn at 10 (solid line), 25, 50, and 100 (dotted line) positions per day. Areas within the solid line are regions for each AIS class where potential AIS disabling events are omitted due to insufficient satellite reception.

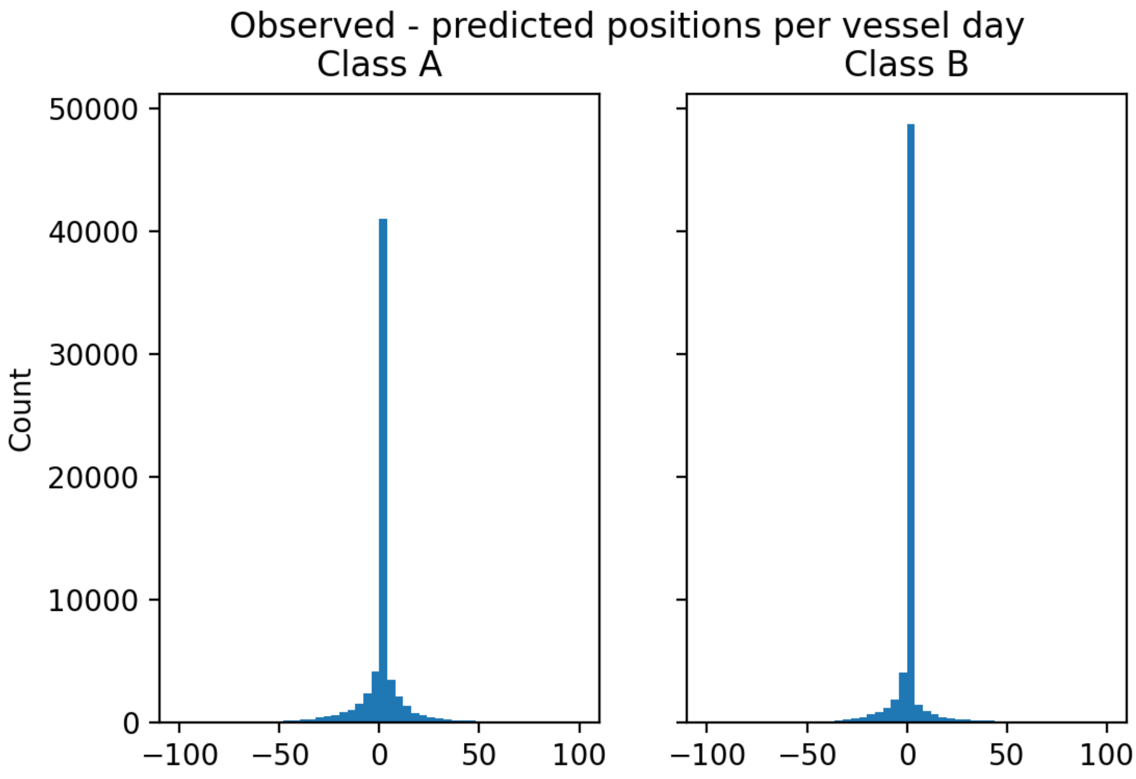

Figure S5: Distribution of AIS reception model residuals by AIS Class. Residuals are normally distributed, with a median residual of 2.3 for Class A and -1.2 for Class B.

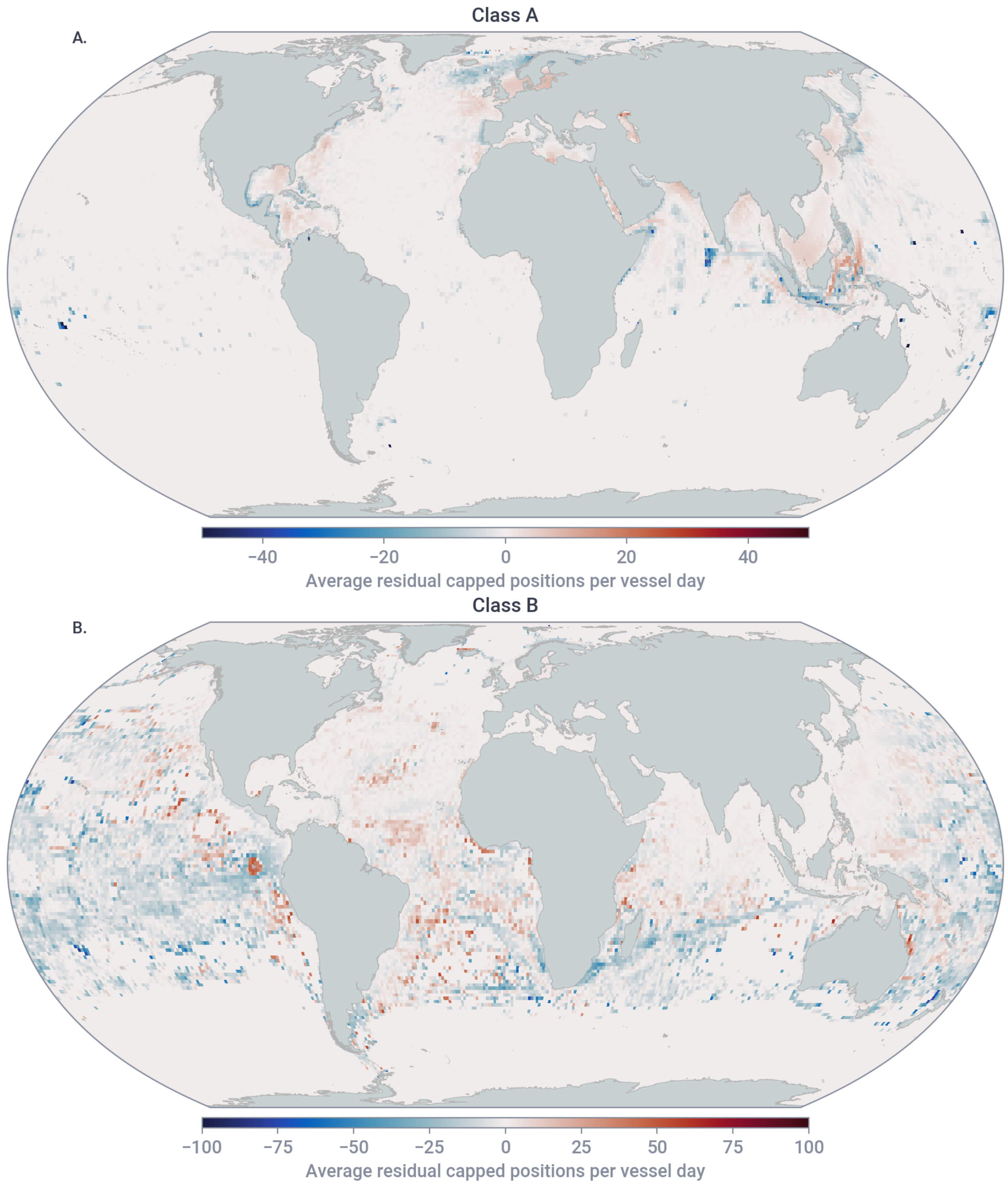

Figure S6: Residuals (observed - predicted) of average reception quality in units of AIS positions ("pings") per vessel day for Class A (A) and Class B (B). Pings per vessel day are capped at 100 and restricted to cells with at least 24 hours of activity in the study period to highlight model performance in areas where AIS reception is of more concern.

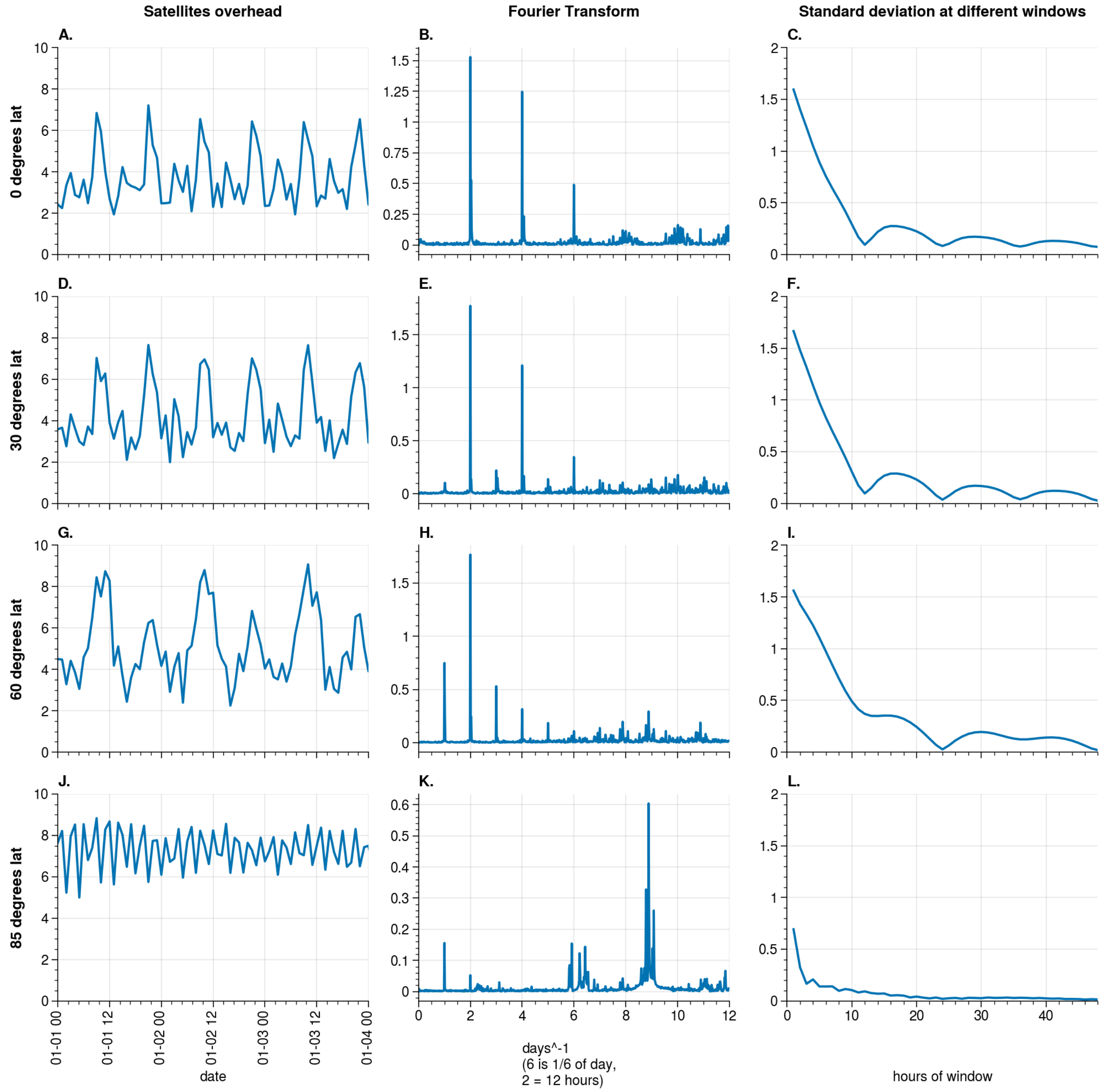

Figure S7. The number of satellites over the horizon at different latitudes varies considerably hour to hour. The number of satellites overhead at each minute of the day is shown across three days at 0° (A), 30° (D), 60° (G), and 85° (J) latitude and 0° longitude. A fast fourier transform of two months of these time series (B,E,H,K) shows that at latitudes of 60° or less, the major peak is at 12 hours (on half a day). Near the poles (85^0 latitude), the periodicity peaks at about 150 minutes, which is roughly the time for a satellite to orbit the earth once, and most of these satellites are in polar orbits and thus pass over the pole. Taking a standard deviation of these time series with different windows shows that the standard deviation of the number of satellites overhead is very high when considering time periods under 12 hours (C,F,I,L).

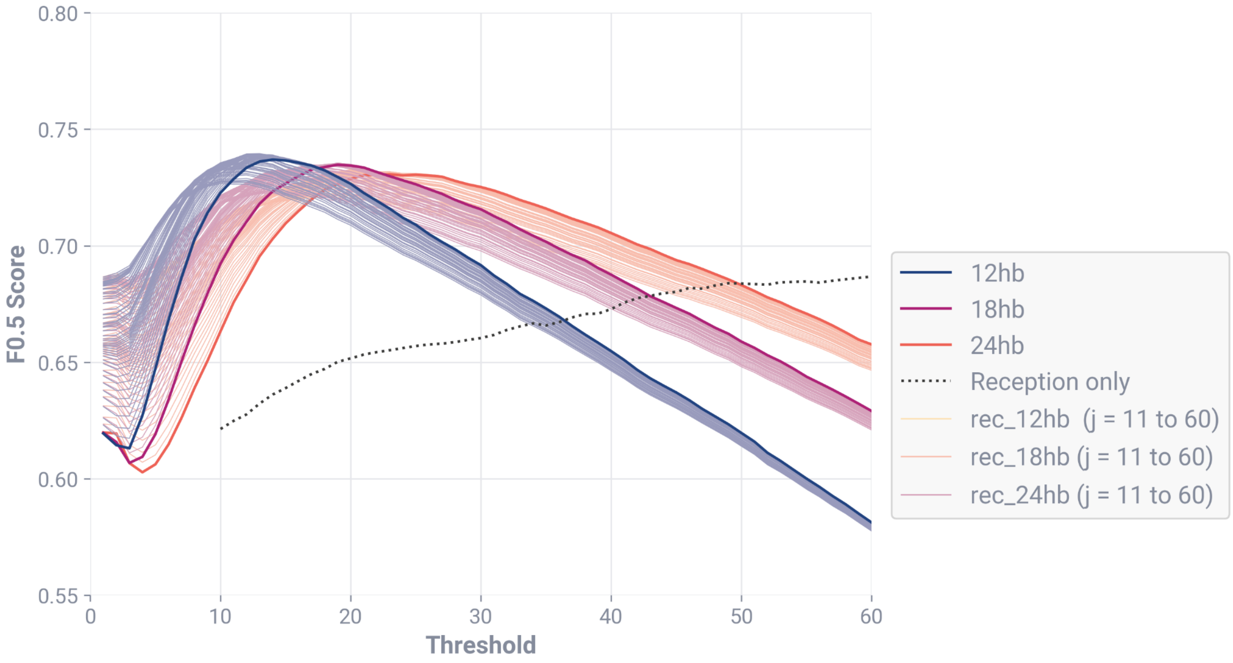

Figure S8: Model performance for each model on the training data set evaluated at all threshold combinations where hb=*hours before and* rec_ *indicates that reception was included in the model (see Table S1 for full model descriptions). The x-axis represents the individual vessel position ping rate threshold (k) for all models except 'Reception only' where it represents reception threshold (j) and has a lower bound of 10 since we are not considering gaps below that threshold. For models that include reception, model performance at each reception threshold (11 ≤* j *≤ 60) is evaluated and plotted separately.*

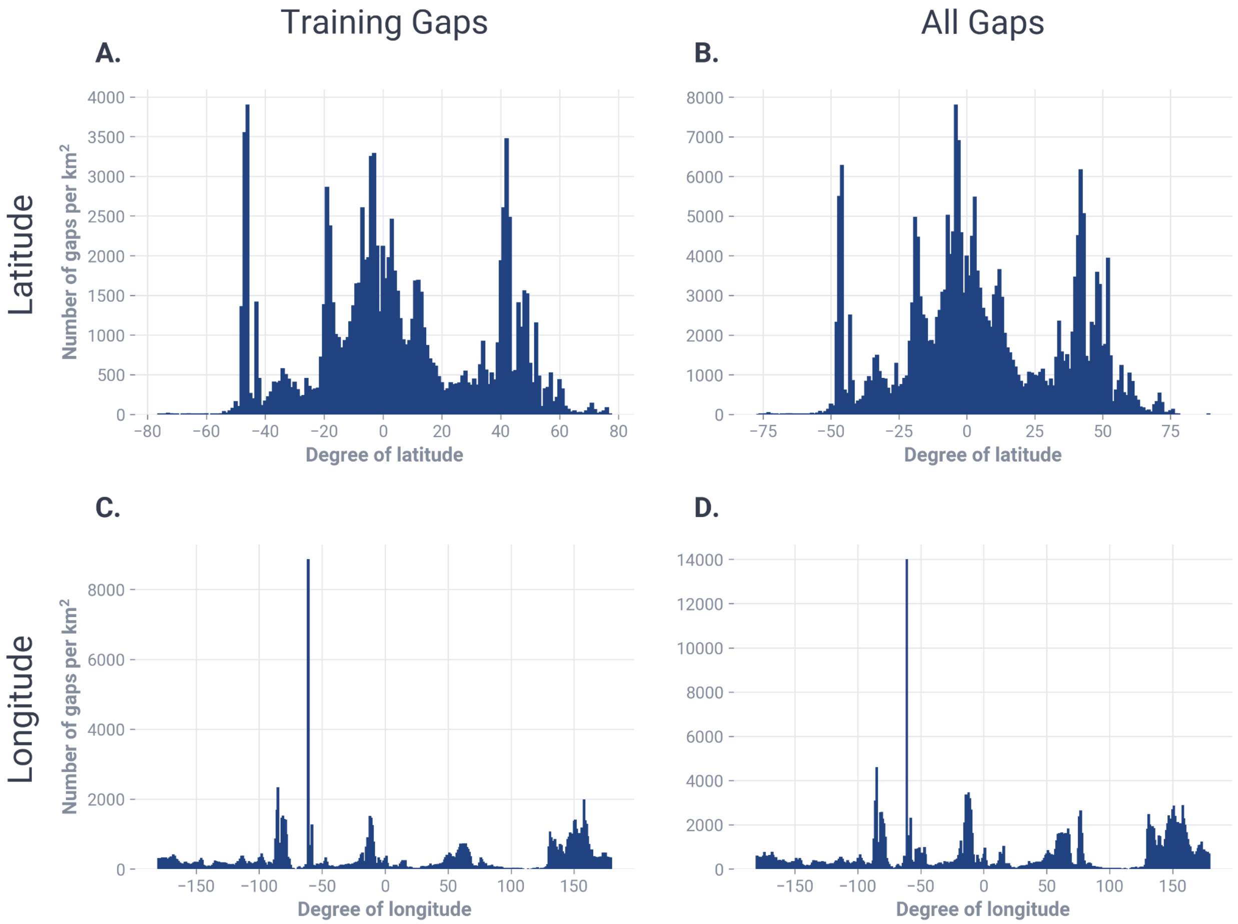

Figure S9: Distribution of training gaps and all gaps across degrees of latitude and longitude. The set training gaps set appears spatially representative of the set of all gaps based on similar distributions and hotspots across latitude and longitude.

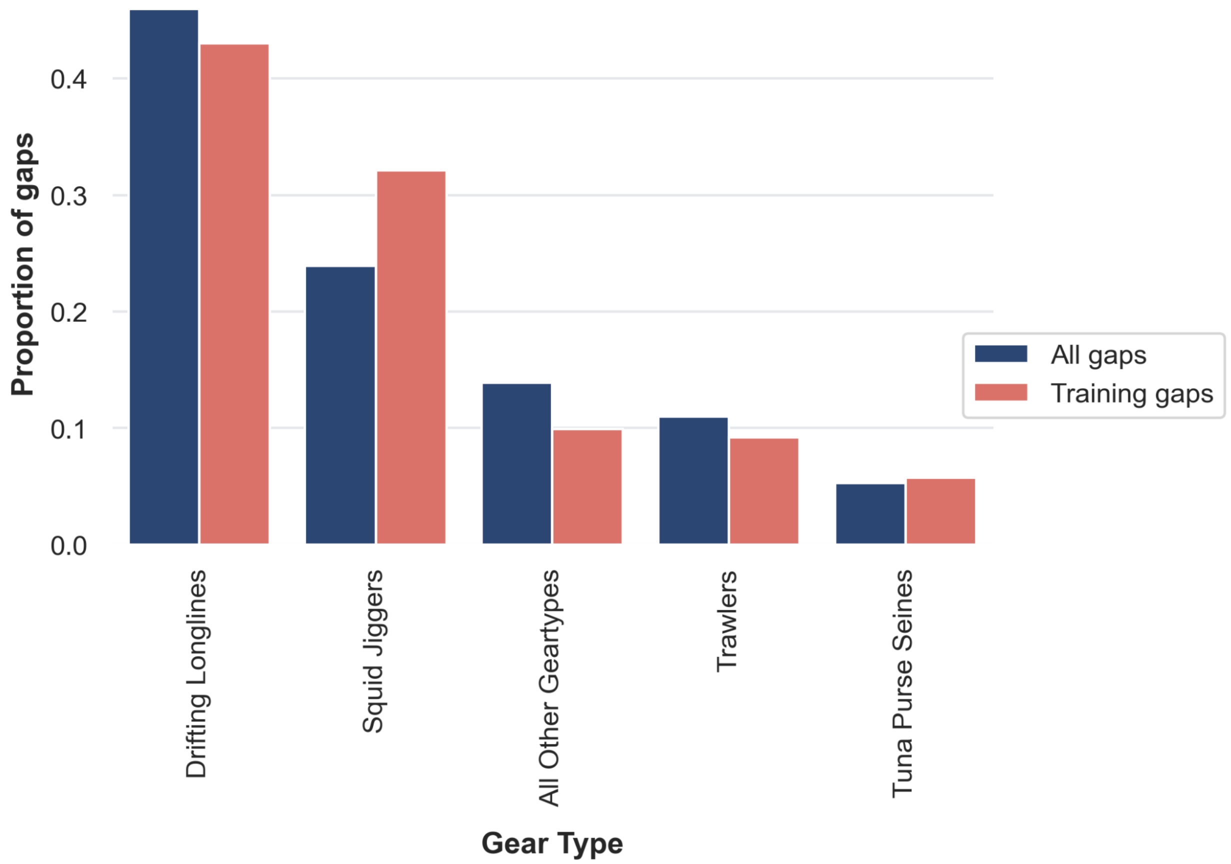

Figure S10: Distribution of training gaps and all gaps by gear type. Both sets have similar proportions of gaps for each gear type, although training sets have a somewhat higher prevalence of squid jiggers and tuna purse seines and somewhat lower prevalence of the other gear type classes.

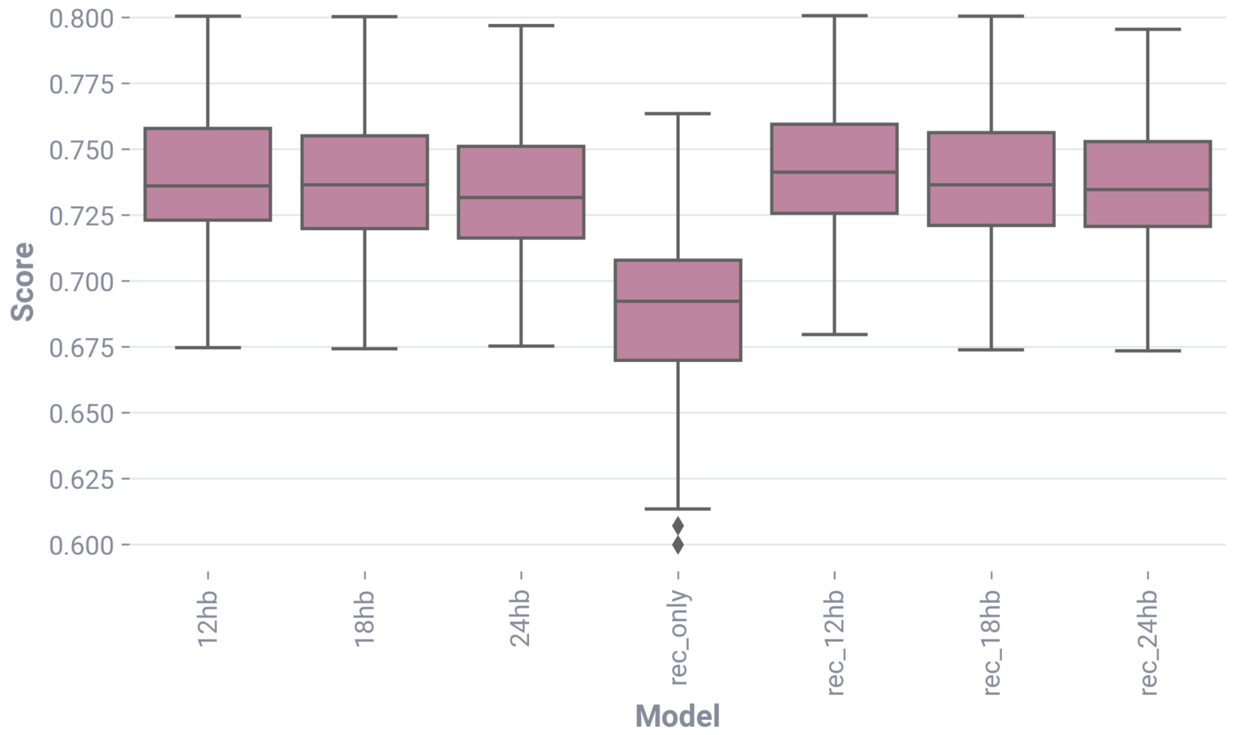

Figure S11: Distribution of cross validation scores for each model. Each boxplot represents the distribution of 100 different model runs in a 10-fold cross validation framework repeated 10 times.

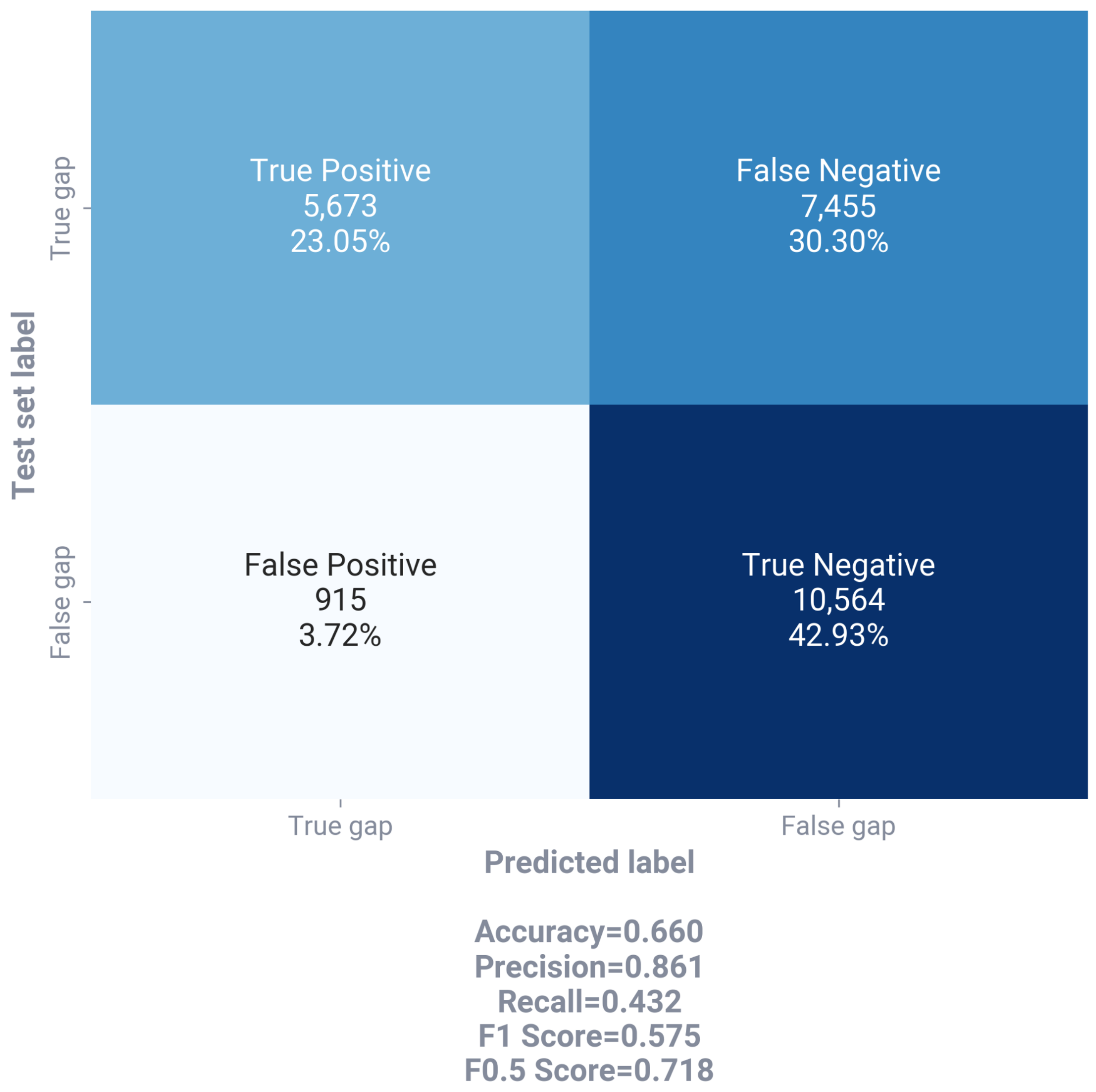

Figure S12: Confusion matrix and accuracy metrics for the optimal model, 12hb >= 14, evaluated on the validation data set.

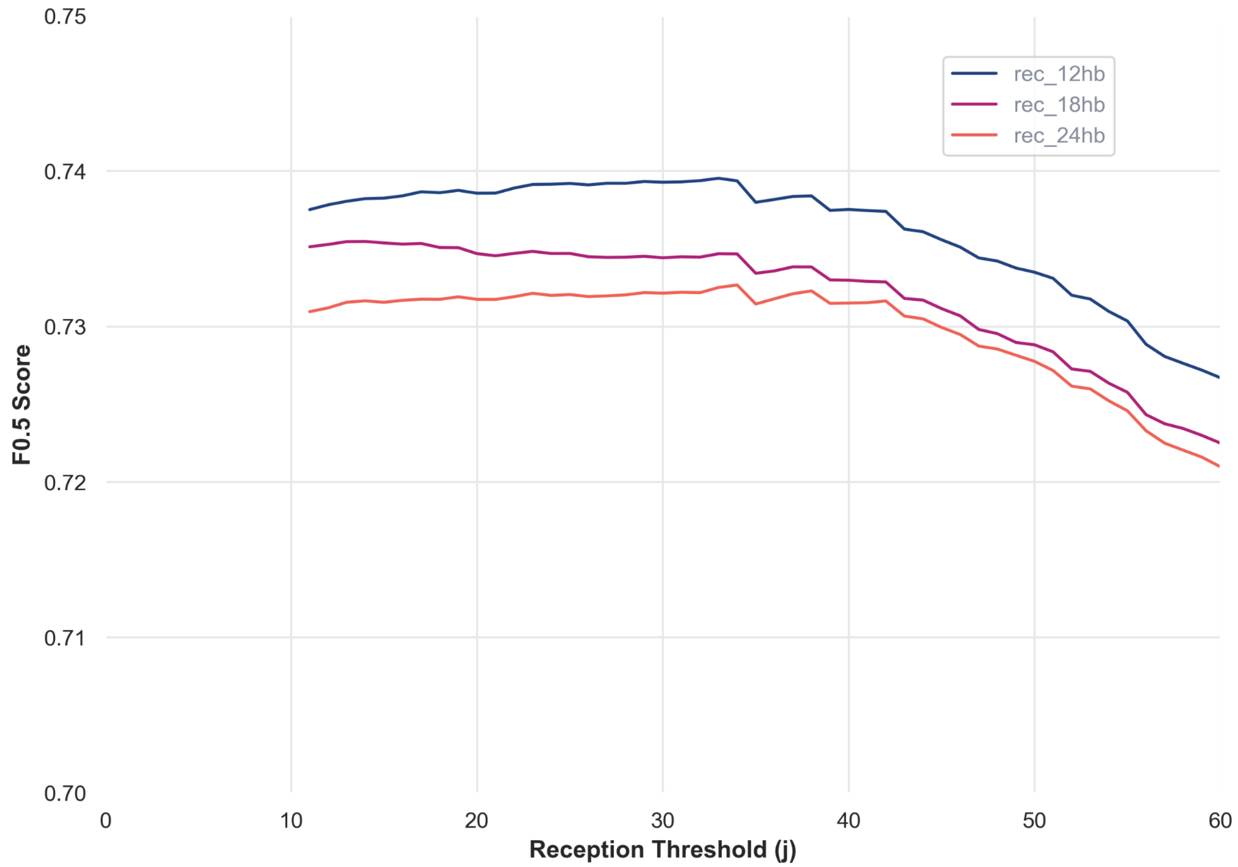

Figure S13: The performance of the optimal model on the training data set at each reception threshold (11 ≤ j ≤ 60). For each reception threshold, the model was evaluated for every individual vessel position ping rate threshold (k) and the maximum F0.5 score was plotted here.

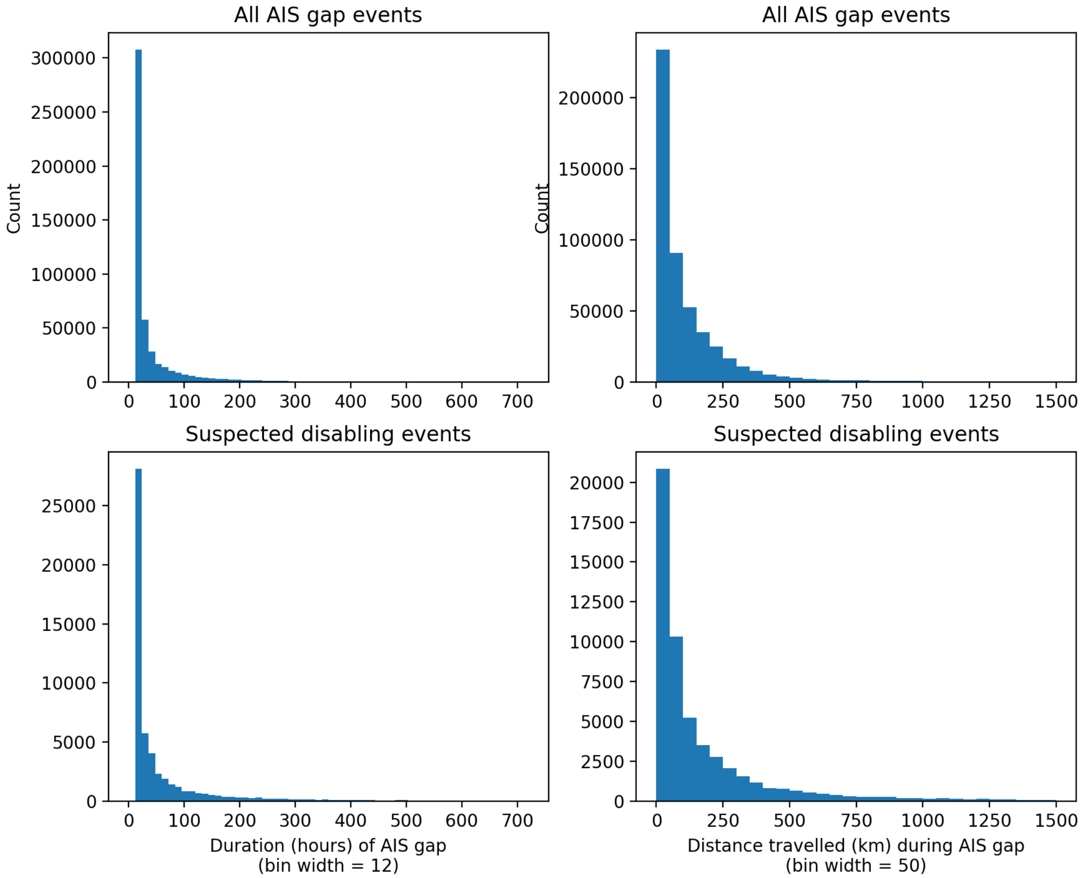

Figure S14: Histograms of the distance and duration of all AIS gap events and suspected disabling events in the study area.

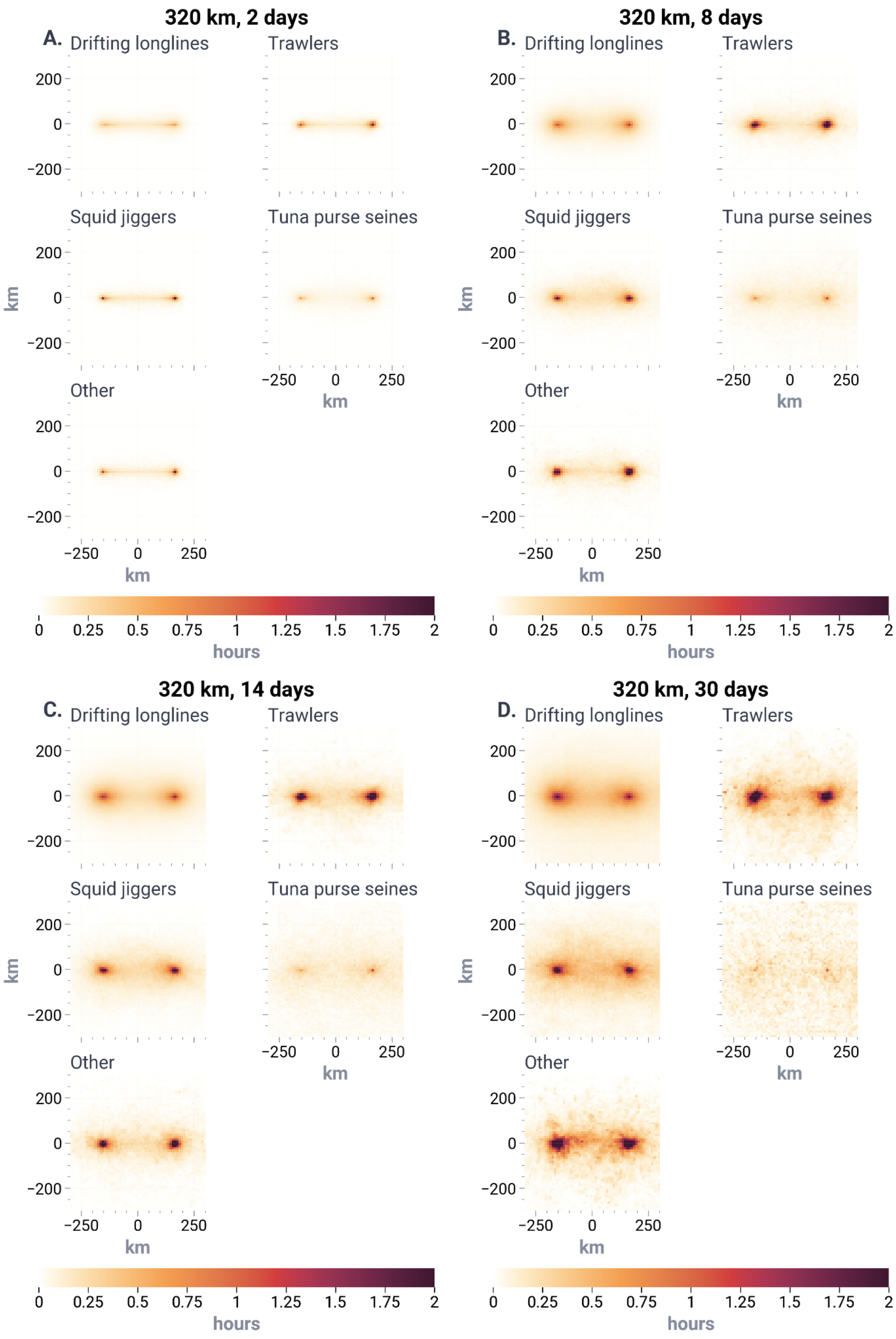

Figure S15: Rasters showing where vessels go between two locations a given distance and duration apart when their AIS is on. To travel between two points 320 km apart in two (A), eight (B), 14 (C), and 30 (D) days, plots show the probable amount of time vessels spent in every location. These distributions provide probabilistic estimates of where vessels may go with their AIS disabled in the rasterized probability method.

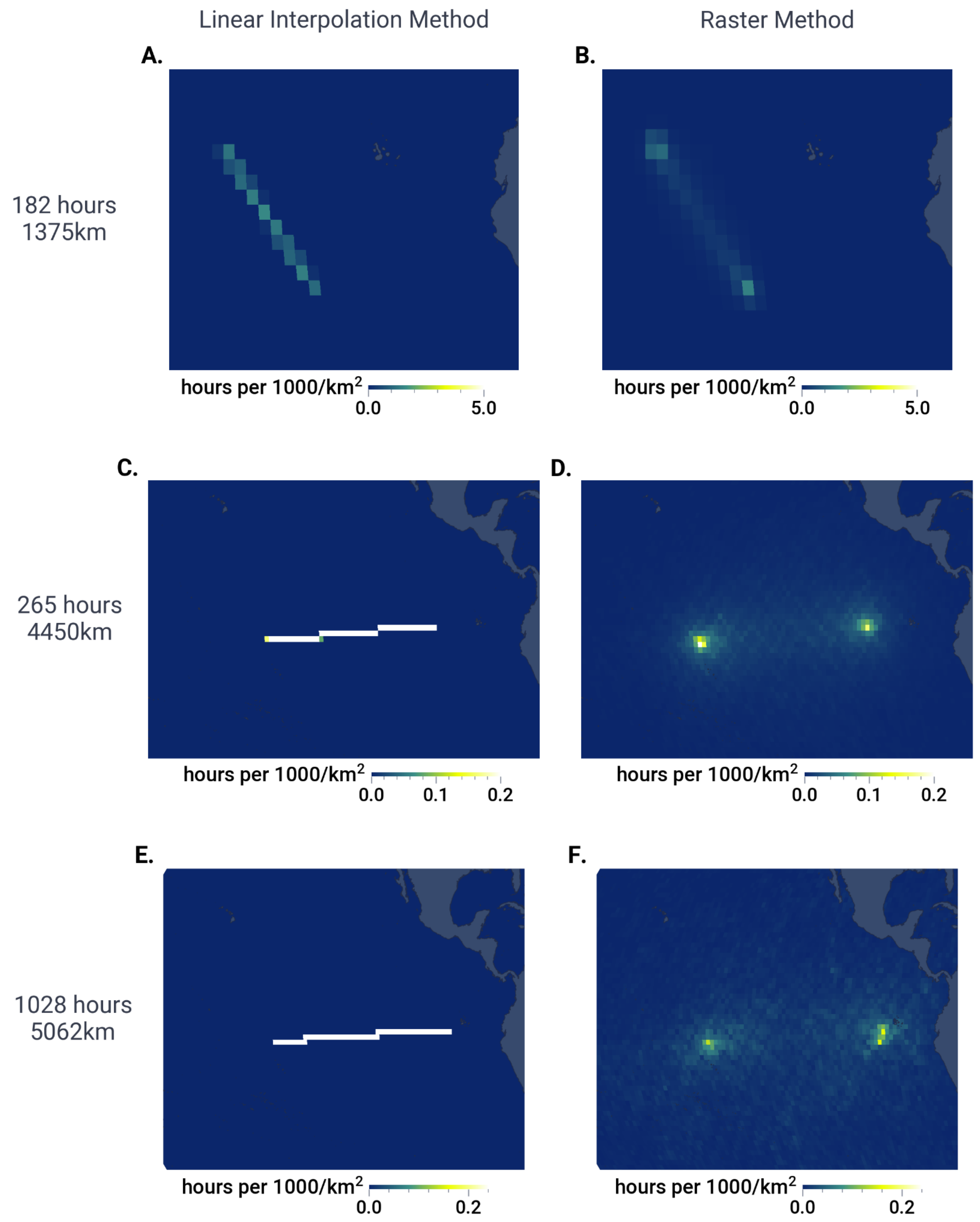

Figure S16: Comparison of three disabling events of varying duration and distance near the Galapagos in the eastern Pacific spatially allocated with the linear interpolation and rasterized

probability methods. Activity with AIS disabled was spatially allocated on a one degree grid using linear interpolation and a probabilistic raster method (See Section 5.2).

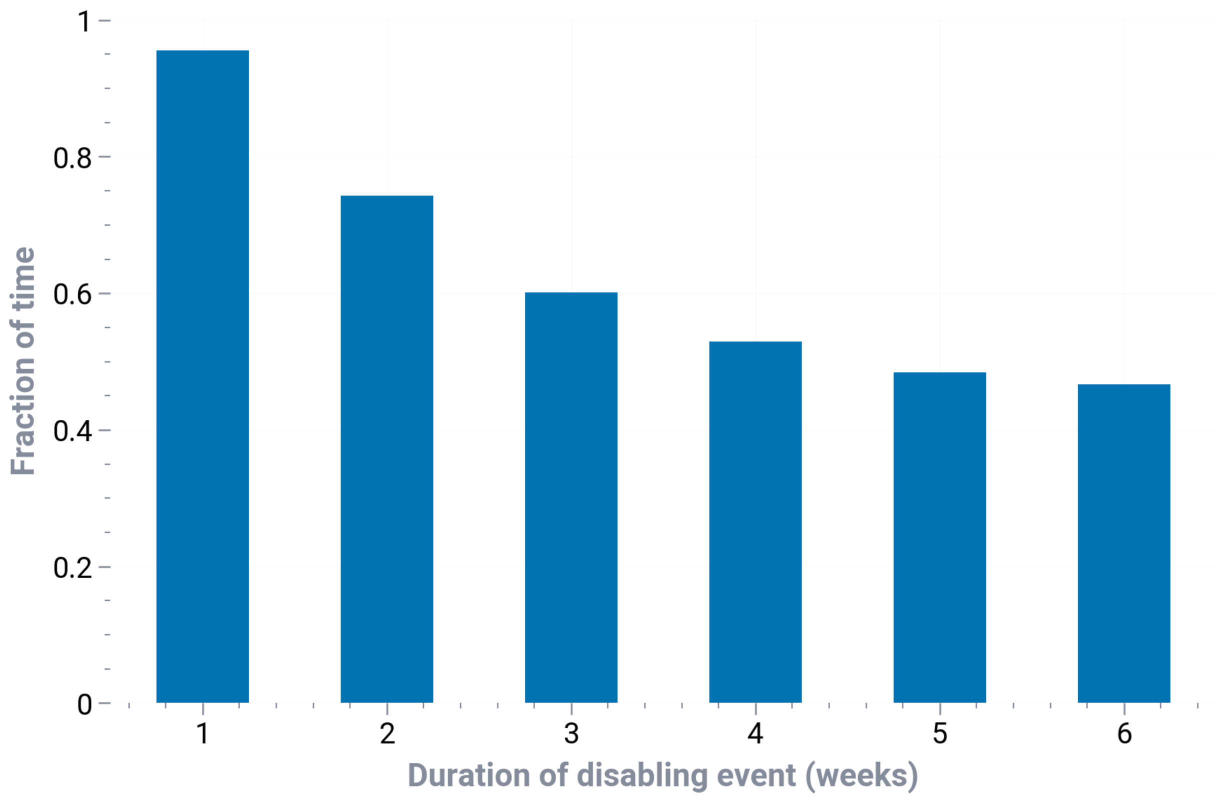

Figure S17: Fraction of spatially allocated time in disabling events (y-axis) based on the rasterized probability method within one degree (111km) of a straight line connecting the start and end of the disabling event, as a function of disabling event duration (x-axis).

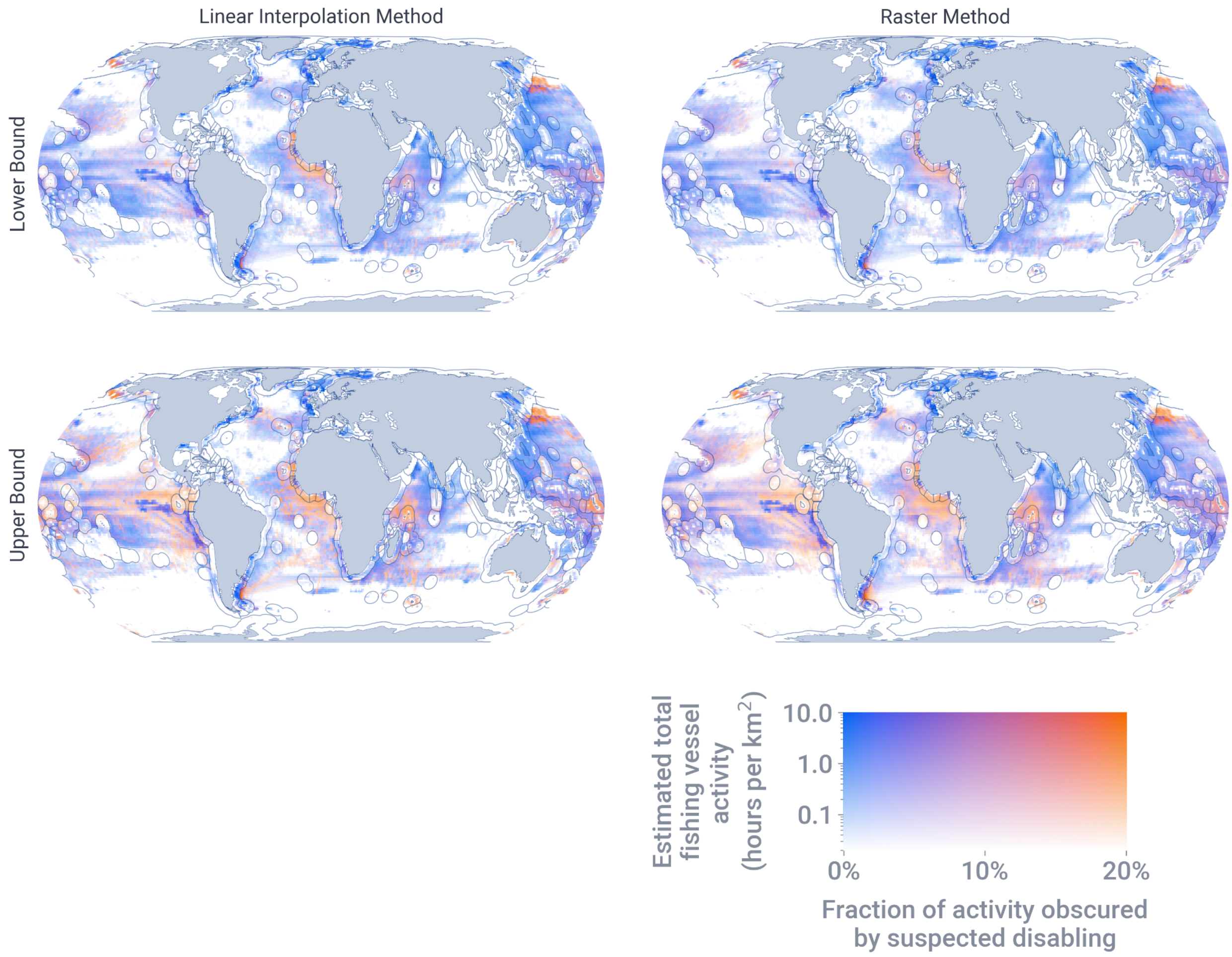

Figure S18: Time disabled at sea for disabling events less than two weeks in duration and all disabling events (lower and upper bounds, respectively) using two different methods of spatially allocating disabling events.

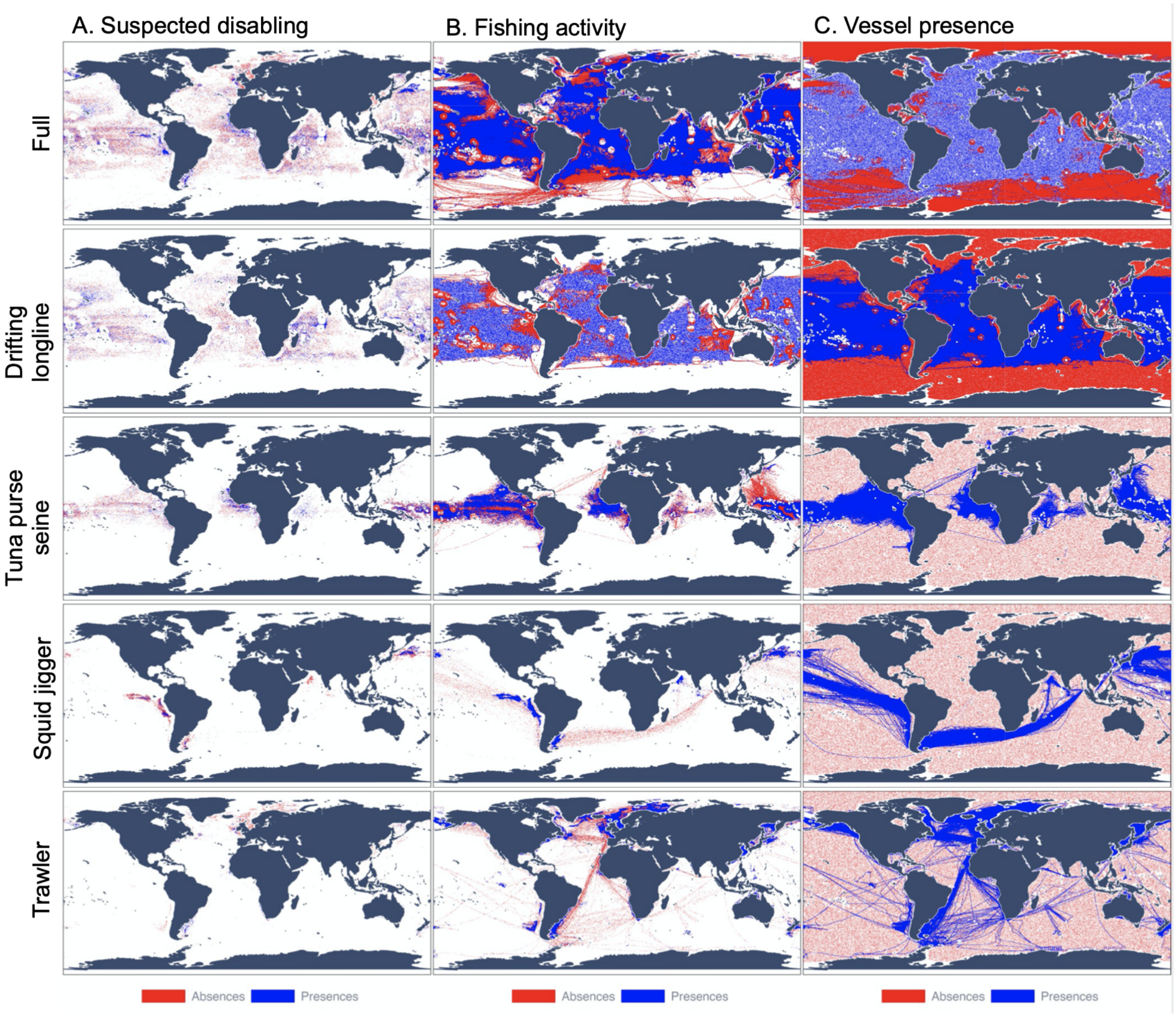

Figure S19. Presences and absences for (A) suspected disabling, (B) fishing activity, and (C) vessel presence models for full, drifting longlines, tuna purse seines, squid jiggers, and trawlers. For suspected disabling, presences are locations with suspected disabling events and absences are locations with fishing activity and no suspected disabling events. For fishing activity, presences are locations with fishing activity and absences are locations with vessel presence and no fishing activity. For vessel presence, presences are locations with vessel presence and absences are background locations with no vessel presence. Presences and absences are shown at the resolution used in model fitting (0.25°).

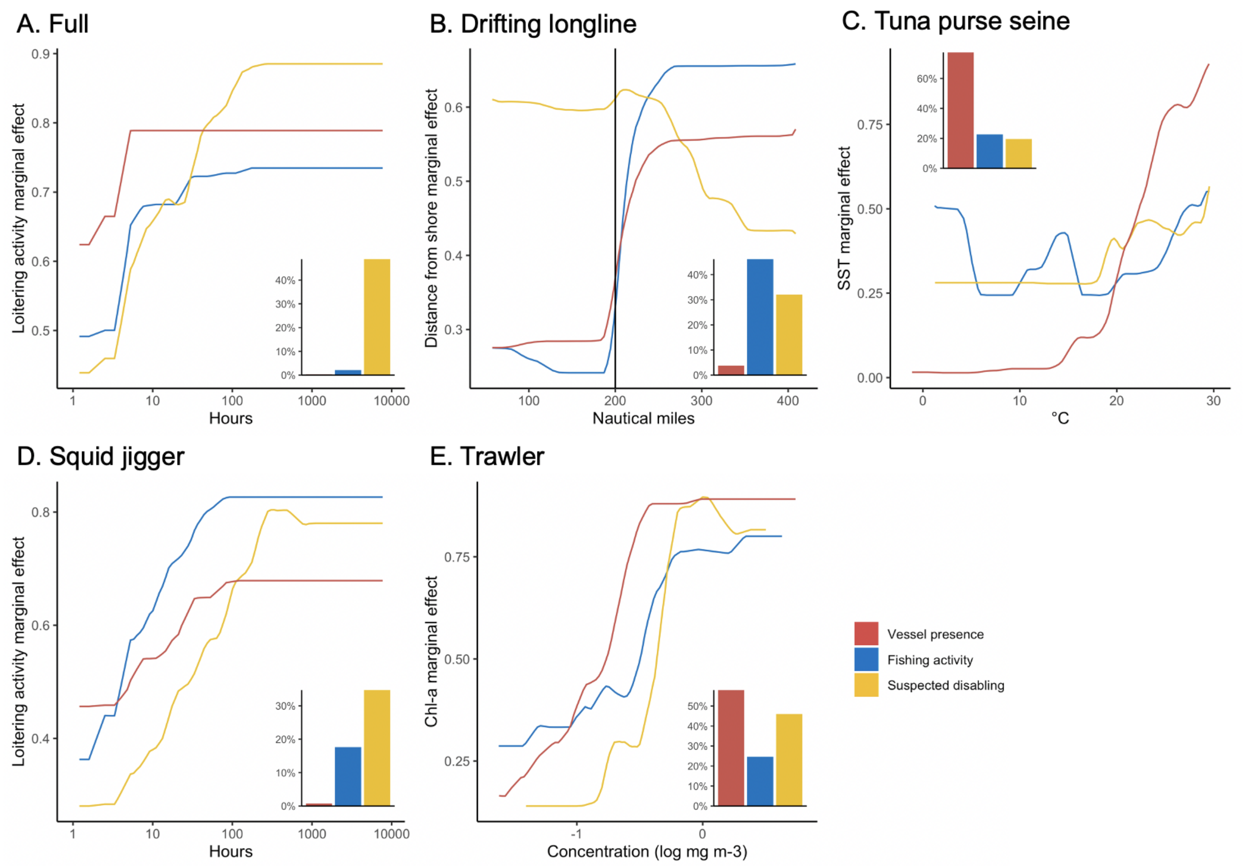

Figure S20. Niche separation of vessel presence, fishing activity, and suspected disabling. Partial dependence plots are shown for each behavior for the most important driver in the each disabling model: (A) loitering activity in the full model, (B) distance from shore in the drifting longline model, (C) sea surface temperature (SST) in the tuna purse seine model, (D) loitering activity in the squid jigger model, and (E) chlorophyll-a (chl-a) in the trawler model. Inset bar plots show the relative importance of drivers in each model.

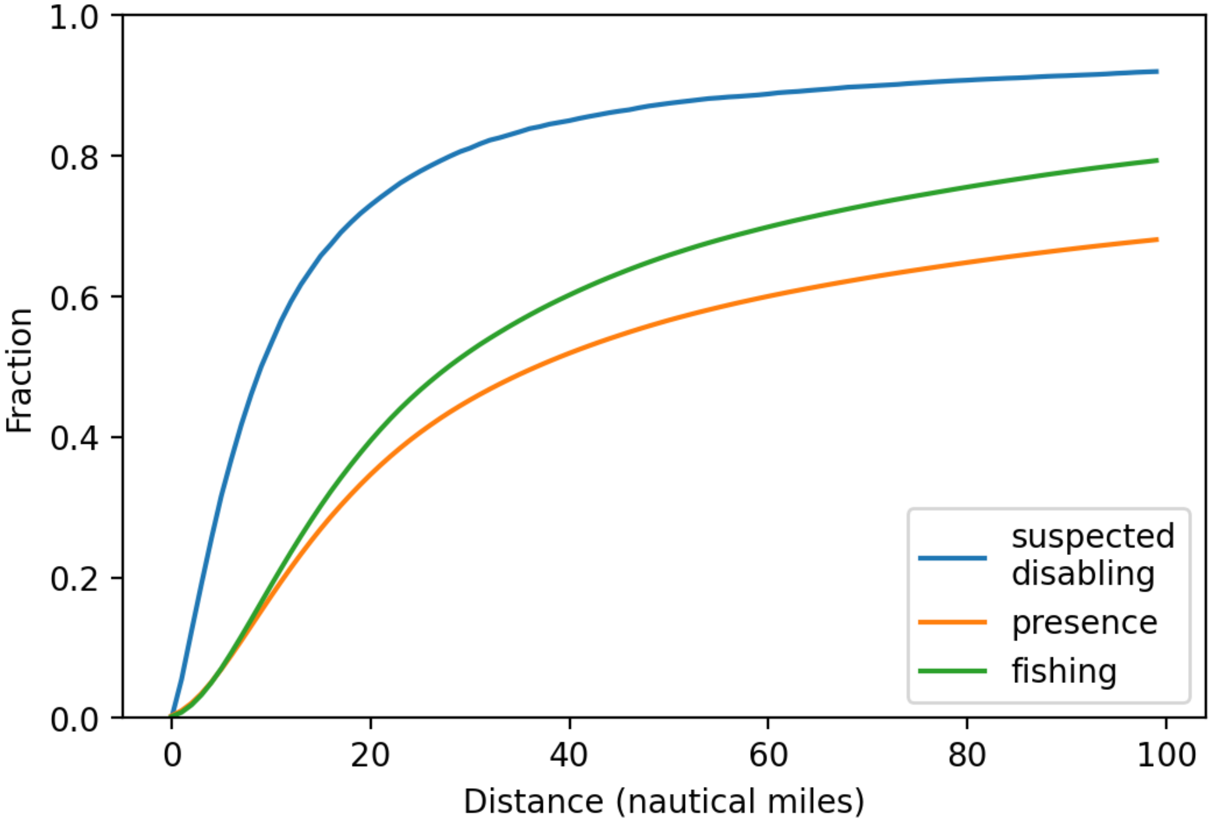

Figure S21: Fraction of all AIS positions ("presence"), fishing positions ("fishing"), and suspected disabling events of squid jigging vessels within a given distance from loitering events.

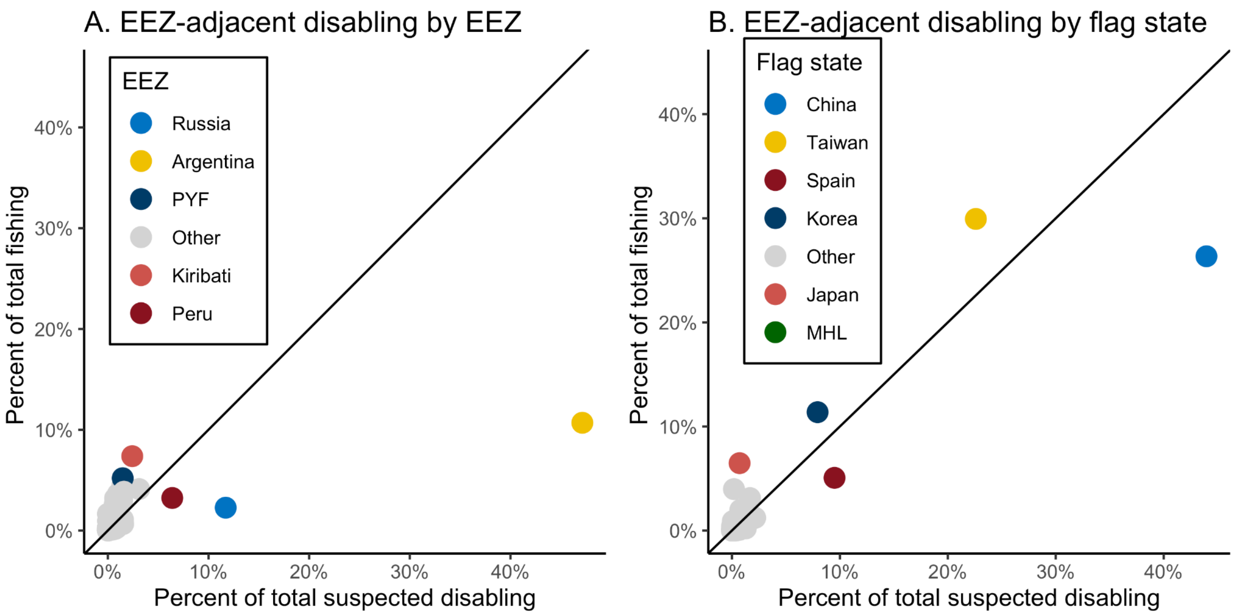

Figure S22. Comparison of Exclusive Economic Zone (EEZ)-adjacent suspected disabling and fishing activity. A & B. The relative percentages of total EEZ-adjacent suspected and fishing activity in each EEZ and by each flag state, respectively. Diagonal lines are 1:1 lines; PYF: French Polynesia.

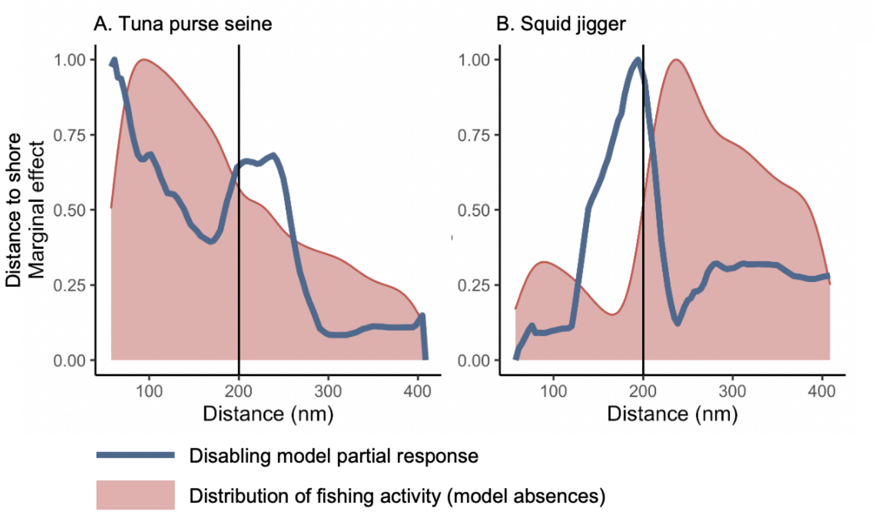

Figure S23. Partial dependence plots for distance to shore for the tuna purse seine (A) and squid jigger models (B). Each plot shows the model marginal response (blue) and distribution of model absences (fishing activity in locations with no disabling, red).

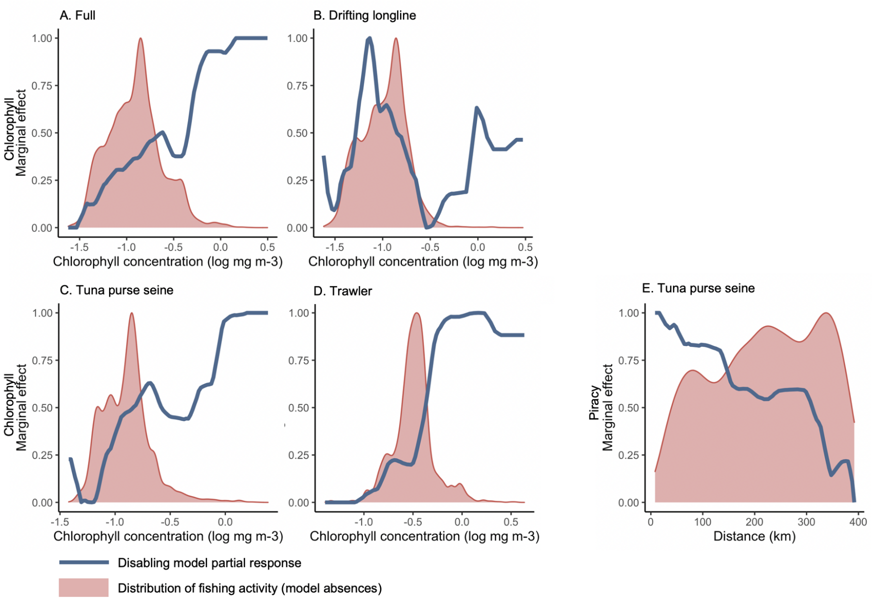

Figure S24. Partial dependence plots for chlorophyll (A-D) for the full (A), drifting longline (B), tuna purse seine (C), and trawler models. (E) shows the partial dependence plot for distance to historical pirate attacks for tuna purse seines (D). Each plot shows the model marginal response (blue) and distribution of model absences (fishing activity in locations with no disabling, red).

Supplementary Tables

Table S1. Descriptions of the rule-based classification models.

Model ID	Description
12hb	number of positions 12 hours before gap >= k*
18hb	number of positions 18 hours before gap >= k*
24hb	number of positions 24 hours before gap >= k*
rec_only	reception at gap start >= j**
rec_12hb	reception at gap start >= j and number of positions 12 hours before gap >= k
rec_18hb	reception at gap start >= j and number of positions 18 hours before gap >= k
rec_24hb	reception at gap start >= j and number of positions 24 hours before gap >= k

**where k is the ping rate threshold to be optimized*

***where j is the reception threshold to be optimized*

Table S2: Cross validation scores for each model. Each score represents the mean of the F0.5 scores of 100 model runs along with the standard error across those runs.

**Model ID**	**Cross Validation Score**	**Standard Error**
12hb	0.739	0.0024
18hb	0.7372	0.00242
24hb	0.7325	0.00242
rec_12hb	0.741	0.0024
rec_18hb	0.7373	0.00241
rec_24hb	0.7349	0.00242
rec_only	0.6886	0.00315

Table S3. Comparison of time lost to suspected disabling by linear interpolation and rasterized probability (in parentheses) using the full duration of suspected disabling events.

Vessel group	AIS disabling events	Time (days) lost to AIS disabling events	Fraction of time lost to AIS disabling events	Average duration (days) of AIS disabling events
**Gear type**				
**Drifting longlines**	18641	83202 (80881)	4.6 (4.5)	4.5 (4.3)
**Squid jiggers**	16021	39524 (37949)	7.2 (7.0)	2.5 (2.4)
**Tuna purse seines**	8620	44735 (39445)	20.6 (18.8)	5.2 (4.6)
**Trawlers**	7913	22823 (21403)	5.0 (4.8)	2.9 (2.7)
**Other geartypes**	4173	16423 (15028)	4.0 (3.7)	3.9 (3.6)
**Flag**				
**China**	15624	45440 (43097)	5.4 (5.1)	2.9 (2.8)
**Chinese Taipei**	12867	43872 (42287)	6.3 (6.2)	3.4 (3.3)
**Spain**	4100	23881 (22807)	13.8 (13.4)	5.8 (5.6)
**United States**	3543	16822 (16197)	8.3 (8.2)	4.8 (4.6)
**Other flags**	19234	76693 (70319)	5.0 (4.6)	4.0 (3.7)
**All**				
**All vessels**	55368	206707 (194707)	6.0 (5.7)	3.7 (3.5)

Table S4 Sources, units and websites for behavioral and environmental drivers. GFW: Global Fishing Watch; PacIOOS: Pacific Islands Ocean Observing System; CMEMS: Copernicus Marine Environment Monitoring Service.

	Driver	Units	Source	URL
**Behavioral**	Loitering	Log hours	GFW	https://globalfishingwatch.org/data/
	Distance to shore	Kilometers	PacIOOS	https://www.pacioos.hawaii.edu/metadata/dist2coast_1deg_ocean.html
	Distance to Marine Protected Area	Meters	World Database on Protected Areas	https://www.protectedplanet.net/
	Distance to reported piracy events	Meters	US National Geospatial-Intelligence Agency	https://www.hsdl.org/?abstract&did=437654
**Environmental**	Sea surface temperature	Celcius	CMEMS	https://view-cmems.mercator-ocean.fr/SST_GLO_SST_L4_NRT_OBSERVATIONS_010_005
	Variability of sea surface temperature across time	Celcius	CMEMS	https://view-cmems.mercator-ocean.fr/SST_GLO_SST_L4_NRT_OBSERVATIONS_010_005
	Chlorophyll-a	Log mg m-3	CMEMS	https://view-cmems.mercator-ocean.fr/OCEANCOLOUR_GLO_CHL_L4_REP_OBSERVATIONS_009_082; https://view-cmems.mercator-ocean.fr/OCEANCOLOUR_GLO_CHL_L4_REP_OBSERVATIONS_009_033
	Eddy kinetic energy	m^2s^{-2}	CMEMS	https://view-cmems.mercator-ocean.fr/SEALEVEL_GLO_PHY_L4_NRT_OBSERVATIONS_008_046; https://view-cmems.mercator-ocean.fr/SEALEVEL_GLO_PHY_L4_NRT_OBSERVATIONS_008_047

Table S5. The number of unique presences for the full and gear-specific models for suspected disabling.

	Suspected disabling
Full	28874
Drifting longline	15233
Squid jigger	3628
Trawler	2342
Tuna purse seine	6642

Table S6. Boosted regression tree model performance metrics. Explained deviance is presented for the final models, while Area Under the Receiver Operator Characteristic Curve (AUC) and True Skill Statistic (TSS) are presented as the mean 75/25 cross-validation across 50 model iterations, ± one standard deviation.

		**75/25 cross-validation**	
	**Explained deviance (%)**	**AUC**	**TSS**
Full	26.21	0.81 ∓ 0.004	0.48 ∓ 0.006
Drifting longline	12.17	0.73 ∓ 0.005	0.33 ∓ 0.009
Squid jigger	47.08	0.91 ∓ 0.006	0.67 ∓ 0.018
Trawler	61.42	0.96 ∓ 0.004	0.79 ∓ 0.013
Tuna purse seine	16.53	0.76 ∓ 0.006	0.4 0.013

REFERENCES AND NOTES

1. E. Druel, K. M. Gjerde, Sustaining marine life beyond boundaries: Options for an implementing agreement for marine biodiversity beyond national jurisdiction under the United Nations Convention on the Law of the Sea. *Mar. Policy* **49**, 90–97 (2014).

2. S. Cullis-Suzuki, D. Pauly, Failing the high seas: A global evaluation of regional fisheries management organizations. *Mar. Policy* **34**, 1036–1042 (2010).

3. D. J. Agnew, J. Pearce, G. Pramod, T. Peatman, R. Watson, J. R. Beddington, T. J. Pitcher, Estimating the worldwide extent of illegal fishing. *PLOS ONE* **4**, e4570 (2009).

4. D. Tickler, J. J. Meeuwig, K. Bryant, F. David, J. A. H. Forrest, E. Gordon, J. J. Larsen, B. Oh, D. Pauly, U. R. Sumaila, D. Zeller, Modern slavery and the race to fish. *Nat. Commun.* **9**, 4643 (2018).

5. M. Mackay, B. D. Hardesty, C. Wilcox, The intersection between illegal fishing, crimes at sea, and social well-being. *Front. Mar. Sci.* **7**, 589000 (2020).

6. D. J. McCauley, P. Woods, B. Sullivan, B. Bergman, C. Jablonicky, A. Roan, M. Hirshfield, K. Boerder, B. Worm, Ending hide and seek at sea. *Science* **351**, 1148–1150 (2016).

7. D. A. Kroodsma, J. Mayorga, T. Hochberg, N. A. Miller, K. Boerder, F. Ferretti, A. Wilson, B. Bergman, T. D. White, B. A. Block, P. Woods, B. Sullivan, C. Costello, B. Worm, Tracking the global footprint of fisheries. *Science* **359**, 904–908 (2018).

8. G. Carmine, J. Mayorga, N. A. Miller, J. Park, P. N. Halpin, G. Ortuño Crespo, H. Österblom, E. Sala, J. Jacquet, Who is the high seas fishing industry? *One Earth* **3**, 730–738 (2020).

9. T. D. White, F. Ferretti, D. A. Kroodsma, E. L. Hazen, A. B. Carlisle, K. L. Scales, S. J. Bograd, B. A. Block, Predicted hotspots of overlap between highly migratory fishes and industrial fishing fleets in the northeast Pacific. *Sci. Adv.* **5**, eaau3761 (2019).

10. D. Bradley, J. Mayorga, D. J. McCauley, R. B. Cabral, P. Douglas, S. D. Gaines, Leveraging satellite technology to create true shark sanctuaries. *Conserv. Lett.* **12**, e12610 (2019).

11. G. G. McDonald, C. Costello, J. Bone, R. B. Cabral, V. Farabee, T. Hochberg, D. Kroodsma, T. Mangin, K. C. Meng, O. Zahn, Satellites can reveal global extent of forced labor in the world's fishing fleet. *Proc. Natl. Acad. Sci. U.S.A.* **118,** e2016238117 (2021).

12. J. Park, J. Lee, K. Seto, T. Hochberg, B. A. Wong, N. A. Miller, K. Takasaki, H. Kubota, Y. Oozeki, S. Doshi, M. Midzik, Q. Hanich, B. Sullivan, P. Woods, D. A. Kroodsma, Illuminating dark fishing fleets in North Korea. *Sci. Adv.* **6**, eabb1197 (2020).

13. SOLAS Chapter V, Regulation 19.2. Carriage requirements for shipborne navigational systems and equipment (2002); https://www.liscr.com/sites/default/files/SOLAS%20V_Reg19.pdf.

14. A.917(22) Guidelines for the onboard operational use of shipborne AIS (A.956(23) (2002); https://puc.overheid.nl/nsi/doc/PUC_1361_14/.

15. J. H. Ford, B. Bergseth, C. Wilcox, Chasing the fish oil—Do bunker vessels hold the key to fisheries crime networks? *Front. Mar. Sci.* **5**, 267 (2018).

16. K. Seto, N. Miller, M. Young, Q. Hanich, Toward transparent governance of transboundary fisheries: The case of Pacific tuna transshipment. *Mar. Policy* **136**, 104200 (2022).

17. N. A. Miller, A. Roan, T. Hochberg, J. Amos, D. A. Kroodsma, Identifying global patterns of transshipment behavior. *Front. Mar. Sci.* **5**, 240 (2018).

18. C. Ewell, S. Cullis-Suzuki, M. Ediger, J. Hocevar, D. Miller, J. Jacquet, Potential ecological and social benefits of a moratorium on transshipment on the high seas. *Mar. Policy* **81**, 293–300 (2017).

19. United Nations Office on Drugs and Crime, Transnational organized crime in the fishing industry—Focus on: Trafficking in persons smuggling of migrants illicit drugs trafficking (2011); www.unodc.org/documents/human-trafficking/Issue_Paper_-_TOC_in_the_Fishing_Industry.pdf.

20. M. Taconet, D. Kroodsma, J. A. Fernandes, Global atlas of AIS-based fishing activity—Challenges and opportunities (2019); https://digitalcommons.fiu.edu/srhreports/iuufishing/iuufishing/84.

21. E. Sala, J. Mayorga, C. Costello, D. Kroodsma, M. L. D. Palomares, D. Pauly, U. R. Sumaila, D. Zeller, The economics of fishing the high seas. *Sci. Adv.* **4**, eaat2504 (2018).

22. Y. Oozeki, D. Inagake, T. Saito, M. Okazaki, I. Fusejima, M. Hotai, T. Watanabe, H. Sugisaki, M. Miyahara, Reliable estimation of IUU fishing catch amounts in the northwestern Pacific adjacent to the Japanese EEZ: Potential for usage of satellite remote sensing images. *Mar. Policy* **88**, 64–74 (2018).

23. A. Doumbouya, O. T. Camara, J. Mamie, J. F. Intchama, A. Jarra, S. Ceesay, A. Guèye, D. Ndiaye, E. Beibou, A. Padilla, D. Belhabib, Assessing the effectiveness of monitoring control and surveillance of illegal fishing: The case of west Africa. *Front. Mar. Sci.* **4**, 50 (2017).

24. D. Belhabib, W. W. L. Cheung, D. Kroodsma, V. W. Y. Lam, P. J. Underwood, J. Virdin, Catching industrial fishing incursions into inshore waters of Africa from space. *Fish Fish.* **21**, 379–392 (2020).

25. R. Hilborn, R. O. Amoroso, C. M. Anderson, J. K. Baum, T. A. Branch, C. Costello, C. L. de Moor, A. Faraj, D. Hively, O. P. Jensen, H. Kurota, L. R. Little, P. Mace, T. McClanahan, M. C. Melnychuk, C. Minto, G. C. Osio, A. M. Parma, M. Pons, S. Segurado, C. S. Szuwalski, J. R. Wilson, Y. Ye, Effective fisheries management instrumental in improving fish stock status. *Proc. Natl. Acad. Sci. U.S.A.* **117**, 2218–2224 (2020).

26. IATTC, The quarterly report July–September 2007 of the Inter-American Tropical Tuna Commission (2007); www.iattc.org/PDFFiles/QuarterlyReports/_English/2007-03_Quarterly-Report.pdf.

27. WCPFC, Western and Central Pacific Fisheries Commission guidelines for the regional observer programme” (2018);

www.wcpfc.int/system/files/WCPFC%20Guidelines%20for%20Regional%20Observer%20Programme_May%202018.pdf.

28. K. Sys, J. J. Poos, J. Van Meensel, H. Polet, J. Buysse, Competitive interactions between two fishing fleets in the North Sea. *ICES J. Mar. Sci.* **73**, 1486–1493 (2016).

29. B. B. Collette, K. E. Carpenter, B. A. Polidoro, M. J. Juan-Jordá, A. Boustany, D. J. Die, C. Elfes, W. Fox, J. Graves, L. R. Harrison, R. McManus, C. V. Minte-Vera, R. Nelson, V. Restrepo, J. Schratwieser, C.-L. Sun, A. Amorim, M. B. Peres, C. Canales, G. Cardenas, S.-K. Chang, W.-C. Chiang, N. de Oliveira Leite, H. Harwell, R. Lessa, F. L. Fredou, H. A. Oxenford, R. Serra, K.-T. Shao, R. Sumaila, S.-P. Wang, R. Watson, E. Yáñez, High value and long life—Double jeopardy for tunas and billfishes. *Science* **333**, 291–292 (2011).

30. R. L. Lewison, L. B. Crowder, B. P. Wallace, J. E. Moore, T. Cox, R. Zydelis, S. McDonald, A. DiMatteo, D. C. Dunn, C. Y. Kot, R. Bjorkland, S. Kelez, C. Soykan, K. R. Stewart, M. Sims, A. Boustany, A. J. Read, P. Halpin, W. J. Nichols, C. Safina, Global patterns of marine mammal, seabird, and sea turtle bycatch reveal taxa-specific and cumulative megafauna hotspots. *Proc. Natl. Acad. Sci. U.S.A.* **111**, 5271–5276 (2014).

31. J. A. Santora, N. J. Mantua, I. D. Schroeder, J. C. Field, E. L. Hazen, S. J. Bograd, W. J. Sydeman, B. K. Wells, J. Calambokidis, L. Saez, D. Lawson, K. A. Forney, Habitat compression and ecosystem shifts as potential links between marine heatwave and record whale entanglements. *Nat. Commun.* **11**, 536 (2020).

32. B. Abrahms, H. Welch, S. Brodie, M. G. Jacox, E. A. Becker, S. J. Bograd, L. M. Irvine, D. M. Palacios, B. R. Mate, E. L. Hazen, Dynamic ensemble models to predict distributions and anthropogenic risk exposure for highly mobile species. *Divers. Distrib.* **25**, 1182–1193 (2019).

33. Oceana, Illegal fishing and human rights abuses at sea: Using technology to highlight suspicious behaviors (2019); 10.31230/osf.io/juh98.

34. M. Carrere, Barco chino que pescaba ilegalmente en Argentina tendría un ‘barco gemelo’ para cometer ilegalidades (Mongabay, 2020); https://es.mongabay.com/2020/05/oceanos-pesca-ilegal-en-argentina/.

35. A. Telesetsky, Laundering fish in the global undercurrents: Illegal, unreported, and unregulated fishing and transnational organized crime. *Ecol. Law Q* **41**, 939–997 (2014).

36. S. Widjaja, T. Long, H. Wirajuda, A. Gusman, S. Juwana, T. Ruchimat, C. Wilcox, Illegal, unreported and unregulated fishing and associated drivers (World Resources Institute, 2019).

37. E. R. Selig, S. Nakayama, C. C. C. Wabnitz, H. Österblom, J. Spijkers, N. A. Miller, J. Bebbington, J. L. Decker Sparks, Revealing global risks of labor abuse and illegal, unreported, and unregulated fishing. *Nat. Commun.* **13**, 1612 (2022).

38. A. J. Ortiz, Agreement on port state measures to prevent, deter and eliminate illegal, unreported and unregulated fishing. *Int. Leg. Mater.* **55**, 1157–1179 (2016).

39. Anti-shipping activity messages (ASAM) database; https://msi.nga.mil/Piracy.

40. G. O. Crespo, D. C. Dunn, G. Reygondeau, K. Boerder, B. Worm, W. Cheung, D. P. Tittensor, P. N. Halpin, The environmental niche of the global high seas pelagic longline fleet. *Sci. Adv.* **4**, eaat3681 (2018).

41. R: A language and environment for statistical computing (R Core Team, 2020); www.R-project.org/.

42. G. Van Rossum, F. L. Drake, *Python 3 Reference Manual* (CreateSpace, 2009).

43. M. D. Buhmann, Radial basis functions. *Acta Numer.* **9**, 1–38 (2000).

44. H. T. Ng, S. M. Wu, T. Briscoe, C. Hadiwinoto, R. H. Susanto, C. Bryant, in *Proceedings of the Eighteenth Conference on Computational Natural Language Learning: Shared Task* (Association for Computational Linguistics, 2014).

45. J. Elith, J. R. Leathwick, T. Hastie, A working guide to boosted regression trees. *J. Anim. Ecol.* **77**, 802–813 (2008).

46. M. A. Cimino, M. Anderson, T. Schramek, S. Merrifield, E. J. Terrill, Towards a fishing pressure prediction system for a western pacific EEZ. *Sci. Rep.* **9**, 461 (2019).

47. M. Barbet-Massin, F. Jiguet, C. H. Albert, W. Thuiller, Selecting pseudo-absences for species distribution models: How, where and how many? *Methods Ecol. Evol.* **3**, 327–338 (2012).
